# Integrative analysis of large scale transcriptome data draws a comprehensive landscape of *Phaeodactylum tricornutum* genome and evolutionary origin of diatoms

**DOI:** 10.1038/s41598-018-23106-x

**Published:** 2018-03-19

**Authors:** Achal Rastogi, Uma Maheswari, Richard G. Dorrell, Fabio Rocha Jimenez Vieira, Florian Maumus, Adam Kustka, James McCarthy, Andy E. Allen, Paul Kersey, Chris Bowler, Leila Tirichine

**Affiliations:** 1 Institut de biologie de l’Ecole normale supérieure (IBENS), Ecole normale supérieure, CNRS, INSERM, PSL Université, 75005 Paris, France; 2 EMBL-EBI, Wellcome Trust Genome Campus, Cambridge, CB10 1 SD United Kingdom; 3 URGI, INRA, Université Paris-Saclay, Versailles, 78026 France; 4 Earth and Environmental Sciences, Rutgers University, 101 Warren Street, 07102 Newark, New Jersey USA; 5 J. Craig Venter Institute, 10355 Science Center Drive, 92121 San Diego, California USA; 6 Integrative Oceanography Division, Scripps Institution of Oceanography, University of California San Diego, La Jolla, California, USA

## Abstract

Diatoms are one of the most successful and ecologically important groups of eukaryotic phytoplankton in the modern ocean. Deciphering their genomes is a key step towards better understanding of their biological innovations, evolutionary origins, and ecological underpinnings. Here, we have used 90 RNA-Seq datasets from different growth conditions combined with published expressed sequence tags and protein sequences from multiple taxa to explore the genome of the model diatom *Phaeodactylum tricornutum*, and introduce 1,489 novel genes. The new annotation additionally permitted the discovery of extensive alternative splicing in diatoms, including intron retention and exon skipping, which increase the diversity of transcripts generated in changing environments. In addition, we have used up-to-date reference sequence libraries to dissect the taxonomic origins of diatom genes. We show that the *P. tricornutum* genome is enriched in lineage-specific genes, with up to 47% of the gene models present only possessing orthologues in other stramenopile groups. Finally, we have performed a comprehensive *de novo* annotation of repetitive elements showing novel classes of transposable elements such as SINE, MITE and TRIM/LARD. This work provides a solid foundation for future studies of diatom gene function, evolution and ecology.

## Introduction

Diatoms are one of the most important and abundant photosynthetic micro-eukaryotes, and contribute annually about 40% of marine primary productivity and 20% of global carbon fixation^1^. Marine diatoms are highly diverse and span a wide range of latitudes, from tropical to polar regions. The diversity of planktonic diatoms was recently estimated using metabarcoding to be around 4,748 operational taxonomic units (OTUs)^2,3^. In addition to performing key biogeochemical functions^4^, marine diatoms are also important for human society, being the anchor of marine food webs and providing high value compounds for pharmaceutical, cosmetic and industrial applications^4^. A deeper understanding of their genomes can therefore provide key insights into their ecology, evolution, and biology.

Complete genome sequences for seven diatom species have been published^5,6^, starting with the centric and pennate diatoms *Thalassiosira pseudonana* and *Phaeodactylum tricornutum*^7^ respectively^8^. Analysis of the *P. tricornutum* genome revealed an evolutionarily chimeric signal with genes apparently derived from red and green algal sources as well as the endosymbiotic host, and from a range of bacteria by lateral gene transfers^7,9,10^, although the exact contributions of different donors to the *P. tricornutum* genome remains debated^6,11,12^, alongside vertically inherited and group-specific genes. Such a diversity of genes has likely provided *P. tricornutum* and diatoms in general with a high degree of metabolic flexibility that has played a major role in determining their success in contemporary oceans.

The availability of a sequenced genome from *P. tricornutum* has also opened the gate for functional genomics studies, e.g., using RNAi, CRISPR, TALEN and bacterial conjugation, which have revealed novel proteins and metabolic capabilities such as the urea cycle, proteins important for iron acquisition, cell cycle progression, as well as red and far/red light sensing^5^ among others. Deeper understanding of the ecology of diatoms and a thorough dissection of the gene repertoire of *P. tricornutum* will however require better information regarding genome composition and gene structure. The first draft of the *P. tricornutum* genome (Phatr1), based on Sanger sequencing, was released in 2005, and contained 588 genome scaffolds totalling 31 Mb. The draft genome was further re-annotated and released as Phatr2 in 2008 with an improved assembly^7^, although many of the gene models contained within this annotation remained incomplete^13^. Subsequently, sequencing technologies have evolved and RNA-Seq has been established as a gold standard for transcriptome investigation, and knowledge has advanced rapidly concerning the roles of DNA methylation and histone modifications on transcriptional regulation. Therefore, we exploited a large set of RNA-Seq reads derived from cells grown in different conditions, in combination with expressed sequence tags (ESTs)^14^ and protein sequences (UniProt), as well as histone post-translational modifications and DNA methylation^15,16^ to re-annotate the *P. tricornutum* genome. This allowed the identification of a significant number of novel transcripts including many encoding reverse transcriptases (Rv) with additional protein domains, suggesting that Rv domains have contributed to genetic innovation in the *P. tricornutum* genome. Such phenomena represent well-known cases of TEs being co-opted by host genomes as means of generating diversity (e.g., adaptive immune systems)^17^. Novel classes of TEs were revealed in this annotation including MITE and SINE elements. We further identified extensive alternative splicing (AS) involved in regulation of gene expression in response to nutrient starvation suggesting that AS is likely to be used by diatoms to cope with environmental changes. We report a conserved epigenetic code, providing the host with different chromatin states involved in transcriptional regulation of genes and TEs. Finally, our work dissected the proposed complex chimeric nature of diatom genomes demonstrating the transfer of green, red and bacterial genes into diatoms, using the greatly expanded genomic and transcriptomic reference libraries that have become available across the tree of life since the publication of the initial genome^10,18^. This resource was released as *Phaeodactylum tricornutum* annotation 3 (Phatr3) and is available to the community on the Ensembl portal (http://protists.ensembl.org/Phaeodactylum_tricornutum/Info/Index).

## Results and Discussion

### Structural re-annotation of the *P. tricornutum* genome reveals numerous new gene models

To generate a new annotation of the *P. tricornutum* nuclear genome, our approach combined high-throughput RNA sequencing data along with ESTs and protein sequences. Several mapping pipelines (see Methods) were used, allowing the prediction of 12,233 gene models with an average gene length of 1,624 bp and ~1.7 exons per gene. The predicted Phatr3 gene models were then compared to the Phatr2 gene models (http://genome.jgi.doe.gov/Phatr2/Phatr2.home.html) and their structural differences can be grouped into the following categories (Table 1, Supplementary Table S1):

1. **New gene models**, genes that are newly discovered and are not present in the Phatr2 gene set.
2. **Unchanged gene models**, gene whose structural annotation remains the same as in Phatr2.
3. **Modified gene models**, genes whose structural annotation has a different 5′ end, 3′ end or both 5′ and 3′ ends with respect to Phatr2. Thirty of these genes with a different N terminus in Phatr3 compared to Phatr2 were validated by their presence within a previously constructed multi-gene reference dataset of aligned plastid-targeted proteins that are well conserved across ochrophyte lineages (Supplementary File S1, panel A)^10^. The N-terminus identified by the Phatr3 gene model in each alignment broadly matches the N-termini identified for orthologous sequences from other ochrophytes (Supplementary File S1, panel B). Furthermore, RT-PCR analysis of six randomly chosen genes within this dataset amplified genes with product length predicted by Phatr3 (Supplementary File S1, panel C).
4. **Merged gene models**, genes that are formed by merging two or more Phatr2 genes into one Phatr3 gene model. Such examples include proteins (e.g., Phatr3_EG02340, Phatr3_EG02341) that merge two domains which are often found together in several other organisms (e.g., a calcium binding EF-hand and a protein kinase). Likewise, a response regulator domain was found to merge with histidine kinase (Phatr3_EG02387), which both are part of the two-component regulatory system widely used in prokaryotes to sense and respond to changes in their environment^19^. Several other examples of merged protein domains of the same pathway can be found in Supplementary Table S1.
5. **Split gene models**, genes that are formed by splitting one Phatr2 gene into two Phatr3 gene models.
6. **Antisense gene models**, genes that are found localized on the antisense strand of previously annotated Phatr2 genes.
7. **Others**, 566 genes which do not fall into any of the categories above. These genes require manual curation which can be achieved through the Web Apollo Portal^20^ which is a real-time annotation tool open to the scientific community for a joint effort to improve the Phatr3 genome annotation.

**Table 1:** Comparison of Phatr3 and Phatr2 annotations. The table presents a summary of Phatr3 and Phatr2 gene comparison statistics. In case of Phatr2-JGI gene models, only filtered models have been considered in each case. The number of genes, in each category, and their corresponding coverage of the genome (in base-pairs) is given. Completeness here refers to the percentage of gene models found to contain both start and stop codons.

Features	Phatr2 (JGI)	Phatr3	Coverage (bp)
Number of genes	10,402	12,233	19,689,514 (71.8%)
New gene models	—	1489	1,755,550 (6.4%)
Unchanged gene models	—	4667	6,900,716 (25%)
Modified 5′ and/or 3′	—	4709	8,484,567 (31%)
Merged gene models	—	194	782,135 (2.9%)
Split gene models	—	262	432,293 (1.6%)
Antisense gene models	—	346	276,422 (1%)
Other gene models	—	566	1,057,831 (3.9%)
Mean gene length	1,474 (bp)	1,624 (bp)	—
Number of exons	18,552	20,885	—
Mean exon length	770 (bp)	886 (bp)	—
Number of introns	8,058	10,932	—
Mean intron length	963 (bp)	142 (bp)	—
Number of intergenic regions	5,157	5,542	—
Mean length of intergenic region	1159 (bp)	307 (bp)	—
Completeness	83.4%	99.1%	—
Number of known protein domains	65,988	74,171	—
Number of genes with known protein domain	9,152	9,910	—

### Assessment of the conservation and complex evolutionary origins of the *P. tricornutum* genome

**Figure 1:**
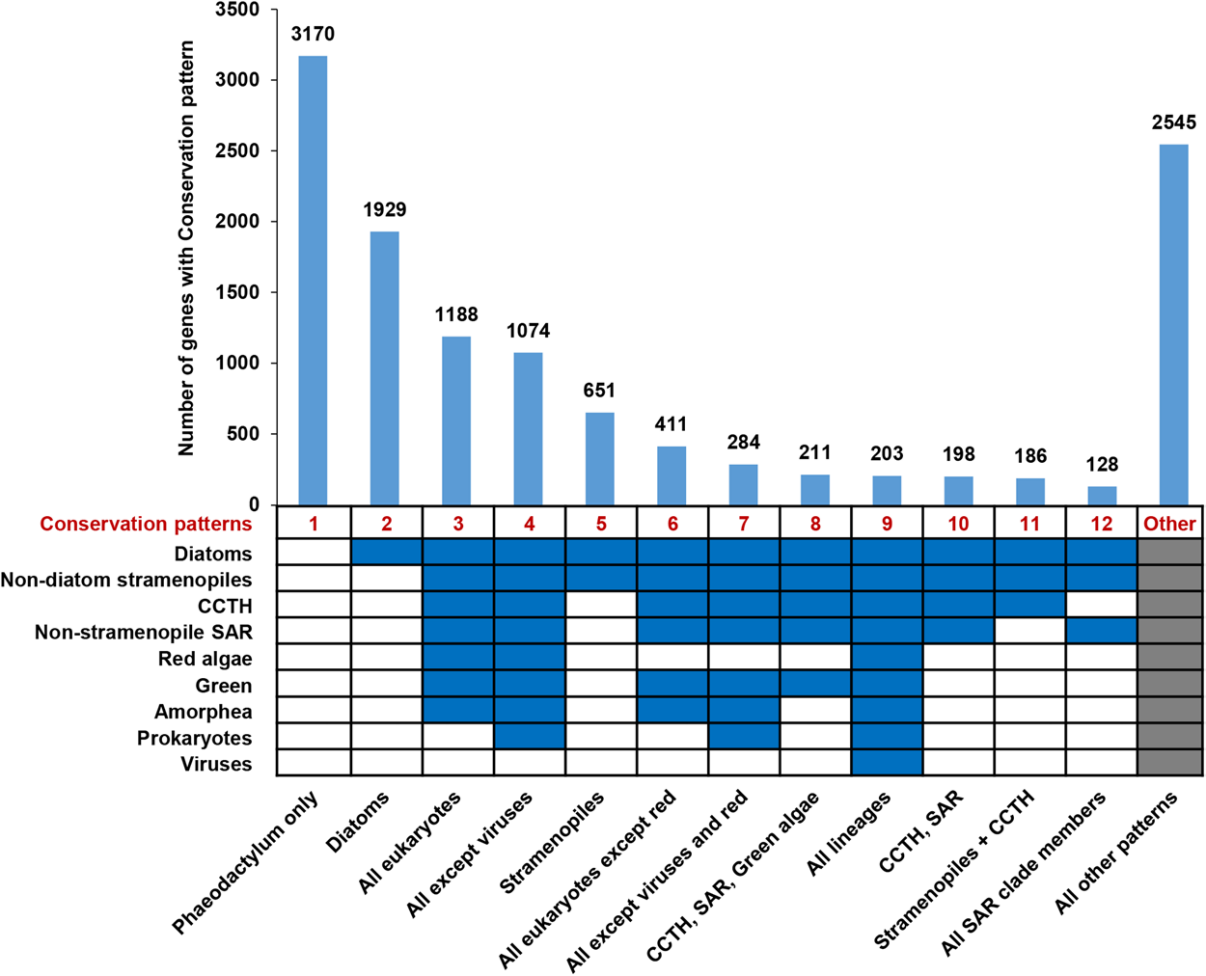
Conserved and group-specific genes in *P. tricornutum*. The chart shows the numbers of different Phatr3 genes shared among different taxonomic groups (Diatoms, Non-diatom stramenopiles, CCTH, Non-stramenopiles SAR, Red algae, Green, Amorphea, Prokaryotes and Viruses) inferring various conservation patterns (indicated at the bottom on each column). The bar-plot shows the number of genes associated with each conservation pattern. Blue color cells, below the bar plot, indicate that orthologues are detected in the corresponding lineage. Only those conservation patterns are discretely indicated which account for at least 100 genes in the Phatr3 genome. Grey cells indicate that either conservation pattern was permitted. SAR refers to the combined clade of stramenopiles, alveolates and rhizaria; CCTH the combined clade of cryptomonads, centrohelids, haptophytes and telonemids; and amorphea refers to the combined clade of opisthokonts, amoebozoa and excavates^21^.

In light of the recent availability of numerous genome and transcriptome sequences from many under-sampled taxa (e.g., red algae) through resources such as the Marine Microbial Eukaryote Transcriptome Sequencing Project^10,18^, we wished to update and re-dissect the proposed complex chimeric nature of the *P. tricornutum* genome. We first aimed to assess the conservation of the *P. tricornutum* proteome across various taxonomic categories, which we grouped together based on recent published phylogenies and taxonomic reviews (Supplementary File S2)^10,21^. From this analysis, a total of 9,008 (74.0%) of the genes within the *P. tricornutum* genome were found shared with at least one other group within the tree of life. This is substantially greater than the ~60% of *P. tricornutum* genes previously identified to have orthologues in other groups^7^, underlining the importance of dataset size and taxonomic sampling when considering gene conservation^11^. Up to 251 different conservation patterns were identified across the entire genome, thirteen of which each accounted for 100 genes or more (Fig. 1, Supplementary Table S2). Many of the genes were found to have broad distributions across the tree of life. We still found, with the expanded dataset, that many genes within the *P. tricornutum* genome have limited evolutionary conservation, with 5750 genes (47.2%) having originated within the recent vertical history of the stramenopile lineage. A total of 3,170 genes (26.0%) were found to be specific to *P. tricornutum*, 1,929 (15.8%) were only shared between *P. tricornutum* and other diatoms, and 651 (5.3%) were only shared with diatoms and other stramenopiles (Fig. 1).

Further, we wished to determine whether the 1,489 novel genes uncovered by Phatr3 differ in terms of evolutionary conservation to those previously identified. While many of the novel genes are specific to *P. tricornutum* (864 genes; 58.0%; Supplementary Fig. S1) or are limited to diatoms (222 genes; 14.9%; Supplementary Fig. S1), 44 genes (13.6%) are shared with at least five other groups, and 4 novel genes are shared with all nine groups considered (Supplementary Fig. S1), including a UvrD-like DNA helicase containing protein (Phatr3_EG00261), CTP biosynthetic process (Phatr3_EG00931), telomere recombination (Phatr3_J11434) genes and a high motility group protein (Phatr3_J1241), confirming that many of these genes are likely to have important biological functions.

In our second analysis, we aimed to reassess the evolutionary origins of the *P. tricornutum* genome (see methods for details). In particular, we wished to validate the presence of genes derived from green algae and from prokaryotes, which have previously been controversial^7,9,11^.

#### Prokaryotic genes

Across the entire dataset, 584 genes yielded top BLAST hits against prokaryotes (Supplementary Table S3), which is similar to the number of prokaryotic genes (587) identified in the initial publication of the *P. tricornutum* genome^7^. These genes resolved with multiple sub-categories, consistent with independent transfer events from different prokaryotic lineages (Supplementary Table S4, Supplementary File S3, panels A-C). Similarly to the initial genome publication, the prokaryotic sub-category that produced the most top hits (235) was the proteobacteria (Supplementary Table S3; Fig. S2A). Nine other sub-categories (cyanobacteria, firmicutes, chlorobi, archaea, actinobacteria, chlamydiae, chloroflexi, the *Deinococcus-Thermus* clade, and planctomycetes) contributed more than ten hits each (Fig. S2A; Supplementary Table S3).

We considered whether the prokaryotic genes present in the *P. tricornutum* genome are recent acquisitions (e.g., species-specific), or are more ancient acquisitions, e.g. by the common ancestors of diatoms, ochrophytes, or earlier (Supplementary File S4; Supplementary Table S3). Instead of originating at a single point, we find that there has been a continuous flux of genes from prokaryotes into different ancestors of *Phaeodactylum* (Supplementary File S4). For example, we detected 22 genes of specifically prokaryotic origin using the full reference dataset, hence were specifically acquired by *P. tricornutum* in its recent evolutionary history. Using the dataset excluding pennate diatoms, we not only detected these 22 genes, but found a further 47 genes, annotated with the full reference dataset as being either of vertical or uncertain origin, which displayed clear prokaryotic affinities (Table S3; Supplementary File S4). These might correspond to genes transferred from prokaryotes into an ancestor of the pennate diatom lineage, prior to the radiation of *Phaeodactylum* from other pennate species in the reference dataset. We similarly detected prokaryotic gene transfers that specifically occurred into common ancestors of the diatom lineage including centric species (133), the ochrophytes (236), the stramenopiles (138) and the SAR clade as a whole (19) (Supplementary File S4).

In addition, different transfer events appear to have occurred with prokaryotes at different points in diatom evolutionary history. For example, 10 of the 15 gene transfers involving members of the *Deinococcus-Thermus* clade are detectable either using the full reference dataset or the dataset excluding pennate diatoms, in contrast to the prokaryotic genes as a whole, which predominantly show more ancient origins. This lineage has not previously been reported to have specifically exchanged genes with an ancestor of *P. tricornutum*^7^. The 10 genes of purported recent origin from the *Deinococcus-Thermus* clade encode metabolic processes including (1) cobalamin-independent synthase which is essential for growth and survival of diatoms in the absence of vitamin B12 (2) proteolysis exemplified by 8 proteins of the subtilase gene family which is important in symbiosis and host pathogen interactions. Interestingly, bacteria of the *Deinococcus* genus were shown to have an expansion of extracellular proteases including subtilases compared to other thermophilic bacteria species^22^. These genes form a cluster on chromosome 17 and an unassembled contig most likely for co-regulation purposes.

#### Red algal genes

**Figure 2:**
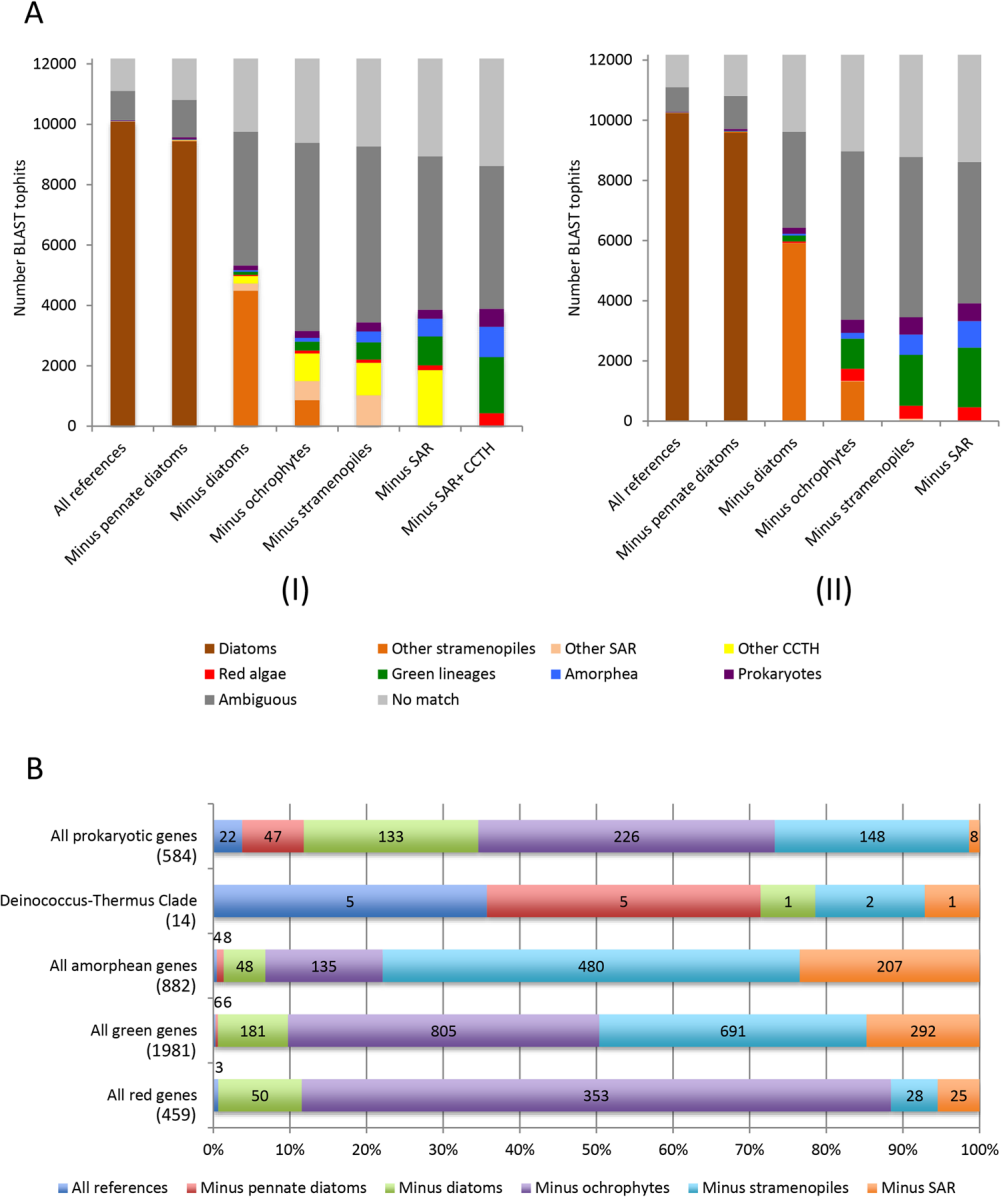
BLAST top hit analysis of *P. tricornutum* genes. (**A**) Comparison of the number of genes of unambiguous taxonomic origin, identified by BLAST top hit analysis of the complete Phatr3 protein annotations against six different reference libraries, constructed using UniRef, jgi genomes and other transcriptome libraries, and six different patterns of taxon inclusion: a library containing all sequences from the tree of life; all sequences except those from pennate diatoms; all except those from diatoms; all except those from stramenopiles; all except those from SAR clade members; and all except those from SAR and CCTH clade members. The right-hand graph (II) shows similar values calculated for reference libraries from which all algae with a suspected history of secondary endosymbiosis other than ochrophytes were removed. Note that in this case a separate value for a reference library excluding SAR and CCTH clade taxa is not provided, as all it is not known when secondary endosymbioses arose within CCTH clade lineages^21^, hence all CCTH clade taxa were excluded from the analysis. (**B**) Comparison of the proportion of genes that yield top hits against five different categories (all prokaryotes, the *Deinococcus-Thermus* clade only, all red algae, and green groups, and all amorphea) following the removal of different groups from the complex algae-free dataset. Total number of genes within each category is indicated beneath it in round brackets. Data labels for each value are provided in the corresponding segment; where the segment is too small to accommodate the corresponding value, the value is placed outside the chart, and shaded to match the color of the segment. The largest value recorded for each gene category corresponds to the most probable evolutionary time point at which genes were acquired; for example, the largest number of genes of red algal affinity were recovered following the removal of all algae with plastids of secondary or higher endosymbiotic derivation from the reference dataset, indicating a large-scale donation of red algal genes into algae with plastids of secondary or higher endosymbiotic derivation.

Across the entire dataset, 459 genes produced BLAST top hits against members of the red algae, consistent with the red algal ancestry of the diatom plastid^4,23^ (Fig. 2A; Supplementary File S3, panel D-E). This is broadly equivalent to the number of red genes identified in previous studies of diatom plastids^24^. The majority of the red genes (353/459) are exposed following the removal of all ochrophyte sequences, with only smaller numbers found following the removal of diatoms (53) or the removal of plastid lacking SAR group members (i.e. oomycetes, labyrinthulomycetes, slopalinids) (53). This is consistent with a red gene transfer happening after the ochrophytes diverged from oomycetes, supporting a late acquisition of the ochrophyte plastid (Supplementary File S3, panel D-E)^10^.

#### Green genes

A total of 1,981 genes generated top BLAST hits from members of the green group (green algae and plants). This is similar in size to the number of green genes (>1,700) identified in previous studies of the origins of diatom groups^24^, and could be consistent with large scale gene transfer between diatom ancestors and green algae (Supplementary Table S3; Supplementary File S3, panels F–H). Some of these genes may be misidentified genes of red algal or other origin, as has been discussed elsewhere^11,12^; however, we believe that many are genuinely of green origin, for two reasons. Firstly, we saw no difference in the representation of red and green algal sub-categories in genes with annotated red or green origin, suggesting these results are not the consequence of taxonomic undersampling (Supplementary Fig. S3). Secondly, green gene transfers appear to have occurred at a different time point to the red algal gene transfers. Although the largest number of putative green genes (805) were identified with the dataset from which all ochrophyte groups were removed (Fig. 2B), nearly as many (691) were identified following the removal of plastid lacking stramenopiles from the dataset (Fig. 2A). Finally, the vast majority of the green gene transfers (1491) involved members of the chlorophyte algae, indicative of one or more gene transfer events from members of this clade, as opposed to vertical inheritance and differential loss of green genes (Supplementary Table S3; Supplementary File S3, panels F–H)^10^.

In summary, the presence of green, red and prokaryotic genes in the *P. tricornutum* genome, which appear to have arisen at different points in its evolutionary history, confirms that it is an evolutionary mosaic, reflects examples of inferred gene transfer events in other major eukaryotic algal lineages^10,25,26^, and underlines the significance of progressive horizontal gene transfer in the evolution and diversification of modern algae^27^.

### Update of the functional annotation of the *P. tricornutum* proteome

We next performed Gene Ontology analysis of the Phatr3 genes using UniProt-GOA (UniProt release 2015_03) and implemented a detailed analysis of their functional domain architecture using Domain Annotation by a Multi-objective Approach (DAMA) and CLADE^28–30^ which is a machine learning method that accurately detect homology relationships between proteins that highly diverged bypassing the limits of current annotation methods. We succeeded to predict the functions and domain architectures of 12,092 genes (~99%), which can be grouped into 5,021 gene families. Among these, the largest gene families are with genes containing reverse transcriptase (Rv) domains (169 genes), RNase H domain (154 genes) and Integrase domain (132 genes). These functional domains often co-localize and are associated with transposable elements. Apart from the latter, protein kinase gene family (115 genes) is also abundant (Supplementary Table S1) within *P. tricornutum*.

Interesting domain architectures were found among the genes that contain either Rv, RNase or H domains alone or together with additional protein domains, including 41 genes possessing Chomo (CHromatin Organization Modifier) domains or 45 cyclins, N/C-terminal domains (Supplementary Table S1). The large number of sequences encoding reverse transcriptase domains in the *P. tricornutum* genome reflects previous studies that suggest these proteins are highly abundant and transcriptionally active in diatoms, implicating a possible role in diatom evolution and adaptation to contemporary environments^31^. The presence of additional protein domains supports previous studies^32^ which suggest that Rv domain-containing proteins might have originated from domesticated retrotransposons that evolved different functions via acquisition of various N- and C-terminal extensions. Besides Rv genes either alone or combined with Chromo and cyclin domains, novel genes are enriched in zinc finger proteins which are one of the most abundant and diverse families of proteins involved in a wide variety of biological functions in eukaryotes^33^. Another common gene family enriched in the newly discovered genes are Ankyrin repeat containing proteins which are known to be involved in mediating protein-protein interactions in diverse biological processes including signal transduction, cell cycle and transcriptional regulation, development and cellular transport mechanisms^34^. Additional significantly enriched gene families include (1) glucosyltransferase genes reflecting the importance of these enzymes in the synthesis of polysaccharides crucial for diatom defense, intercellular communication, bacterial symbiosis, aggregation and biofilm formation^35,36^, (2) autophagy components pathway important in algae for degradation and recycling of cell materials upon exposure to nutrient starvation as well as clearing damaged organelles or toxic cellular components generated during stress to maintain cellular homeostasis^37^.

We further aimed to determine whether genes with different levels of conservation, as determined by our analysis (Supplementary Table S2), have different functional properties in the *P. tricornutum* genome. We performed GO enrichment analysis on four different biological categories of genes, as defined by the presence or absence of orthologues in other lineages by reciprocal best BLAST hit (RbH) analysis (Fig. 1, Supplementary Fig S5, Supplementary Table S5). Interestingly, a high number of Pt-specific genes encode the DNA integration GO category, which may indicate a permissive way for integration of genetic material from diverse sources, thus creating novel genetic diversity. Regulation of transcription is one of the important functional categories of diatom-specific genes, perhaps reflecting differences in promoter architecture compared to other eukaryote.

Next, we used ASAFind and HECTAR to predict the sub-cellular targeting of the Phatr3 proteome (Supplementary Table S6). Across Phatr3, 3196 proteins (26.3% total; Supplementary Fig. S6A) were predicted to have a targeting sequence of some description by ASAFind, and 4067 proteins (33.3%; Supplementary Fig. S5B) were predicted to have a targeting sequence by HECTAR. We then compared the Phatr3 proteome targeting predictions with the new Phatr3 gene models and Phatr2 JGI gene models. A greater proportion of the Phatr3 genes, including the new gene models, contain complete N-termini than Phatr2 (Supplementary Fig. S6), reflecting that Phatr3 genes are better annotated structurally (Supplementary File S1). Several of the new gene models with defined targeting preferences had functions consistent with their localization: for example, we identified through ASAFind a plastid-targeted serine acetyltransferase (Phatr3_EGO1815), which forms an essential component of plastid cysteine synthesis pathways in ochrophytes^10,38^.

**Figure 3:**
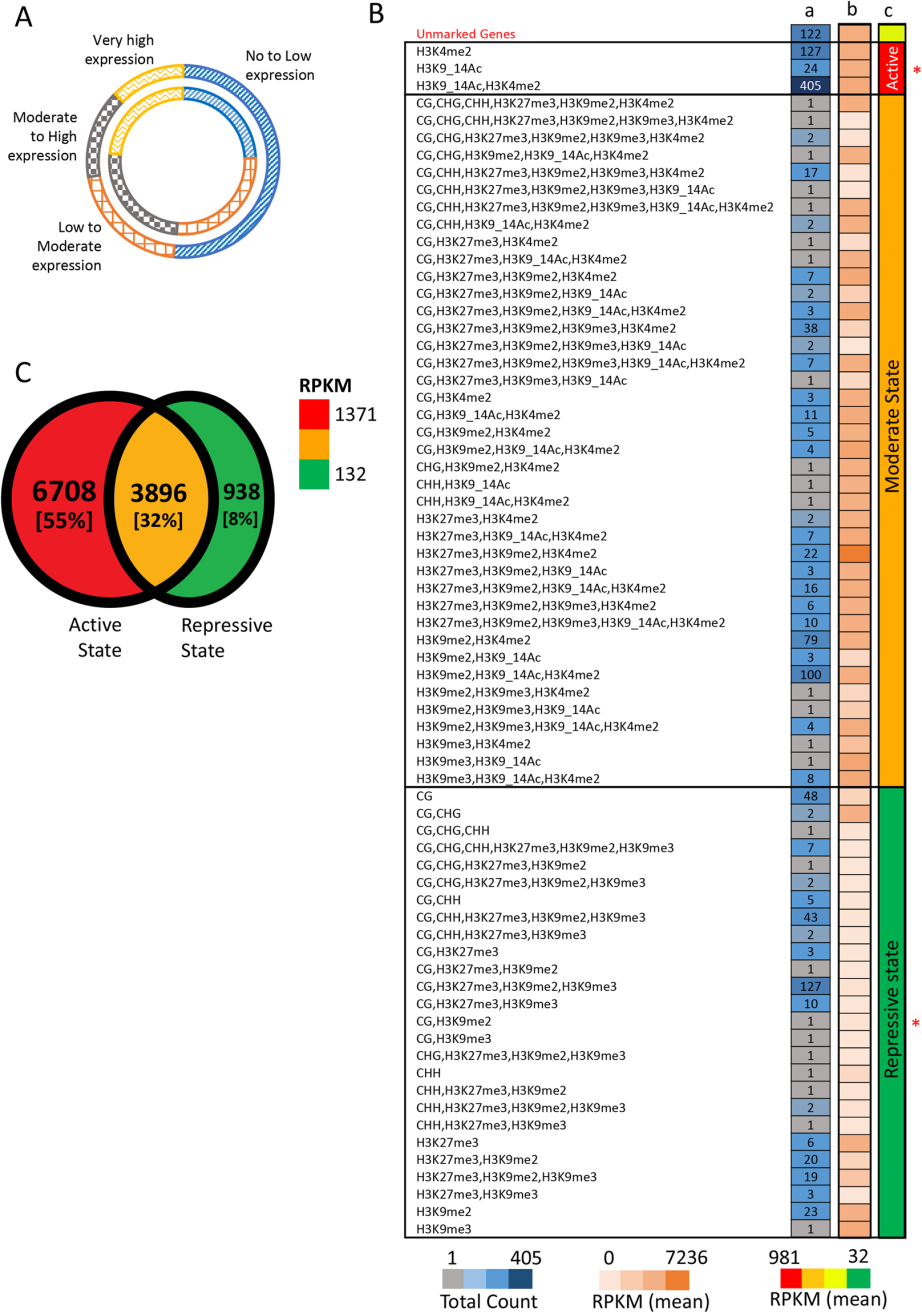
Dissecting the functional characteristics of Phatr3 proteome. (**A**) Comparison of the expression profile of the proportion of new genes (outer circle) with that of the proportion of unchanged gene models (inner circle). The expression profiling of all the Phatr3 genes are done based on quartile method, where first and last quartile reflects genes with no/low expression and very high expression, respectively. (**B**) and (**C**) summarize various epigenetic modification and regulatory effects on Phatr3 gene models and only new gene models in Phatr3. (**B**) Specifies the regulatory effects of the co-localization of different modifications/marks on the novel genes. The number of genes corresponding to each combination of co-localizing marks is provided in column a. The average expression of these genes is represented as a heat map in column b. Compared to the unmarked genes the regulatory effect of the co-localization of different epigenetic modifications is seen to maintain three chromatin states, represented with column c with few exceptions with expression that can be higher or lower than expected. This cases are likely due to the presence of additional active or repressive marks that were not studied. *Indicates where the average expression (normalized DESeq counts) is significantly different (two sample T-test with unequal variance, P-value < 0.002), compared to the expression of unmarked genes. Out of 1,489 genes, 1,388 are considered in the analysis, as expression for the other 101 genes was not calculable without errors. (**C**) Different chromatin states maintained genome wide, based on the association of protein coding genes with repressive, active or both repressive and active chromatin modifiers (Repressive modifications: CG, CHG, CHH, H3K27me3, H3K9me2, H3K9me3; Active modifications: H3K4me2, H3K9_14Ac). Numbers and percentages (in square brackets) in the Venn diagram reflects the absolute number of genes and the relative percentage of the total Phatr3 genes.

We also considered the expression dynamics of each gene using quartile approach. Most of the novel genes (~70%) are expressed at below the median level inferred for all other genes in the genome (Fig. 3A) and are mostly specific to *P. tricornutum* (Supplementary Fig. S1). We then compared chromatin marks associated with new versus unchanged Phatr3 gene models and found that the proportion of DNA methylated genes within new gene models (30%, 448 genes) was found to clearly delineate the proportion within unchanged gene models (9%, 4,667 genes) (Supplementary Table S1). The majority of these are at least methylated in CG context, which is in line with previous work^15^. Thus, along with boosting the functional content of the genome, newly discovered genes will certainly expand our capacity to understand the role of DNA methylation in the regulation of the *P. tricornutum* genome. Broadly, among the 1,489 new genes, 1,360 (~91%) genes are marked by at least one of the epigenetic modifications studied previously (Supplementary Table S1, Supplementary Fig. 3B).

Finally, we considered an update on the distribution of DNA methylation and post-translational modifications of histone H3 (PTMs) across the *P. tricornutum* genome, following previous work^15,16^. Up to 11534 genes (~95%) within Phatr3 were found to be associated to the studied H3 PTMs or to DNA methylation (Fig. 3C, Supplementary Table S1), either alone or in combination supporting the conservation of an epigenetic code which is critical for transcriptional regulation of genomes and will be useful to decipher the role of epigenetics in underpinning the ecological success of diatoms.

### Intron retention is prominent in *P. tricornutum* and regulates genes under fluctuating environmental conditions

The functional and regulatory capacity of eukaryotic genomes is greatly influenced by alternative splicing of precursor RNAs. Intron-retention (IR) is a major constituent of the alternative splicing code in plants and unicellular eukaryotes^39^, whereas exon-skipping (ES) is prominent within members of the metazoan clade; however, the broader evolutionary histories of both processes across the eukaryotes remains unclear^40,41^. Therefore, and considering the position of diatoms in the tree of life, we investigated the nature and dynamics of both processes within the Phatr3 genome.

First, we mapped the distribution and dynamics of introns in the *P. tricornutum* genome. In total, we found 8,646 introns (on average 0.7 introns per gene) from which >99.8% include canonical splice-sites (Acceptor Sites: AG/CT and Donor sites: GT/AC). Up to 4,014 (~33%) of the Phatr3 genes were predicted to contain only one intron, while 1,730 (~14%) genes contain more than one intron and 6,434 (~53%) are predicted to be intron-less. The low density of introns and small intron size observed in *P. tricornutum* is similar to many unicellular eukaryotes, including the related diatoms *T. pseudonana* and *Fragilariopsis cylindrus*^40,42,43^, which might mirror genome size, or enhance transcriptional efficiency or splicing accuracy in metabolically fluctuating environments^44,45^.

**Figure 4:**
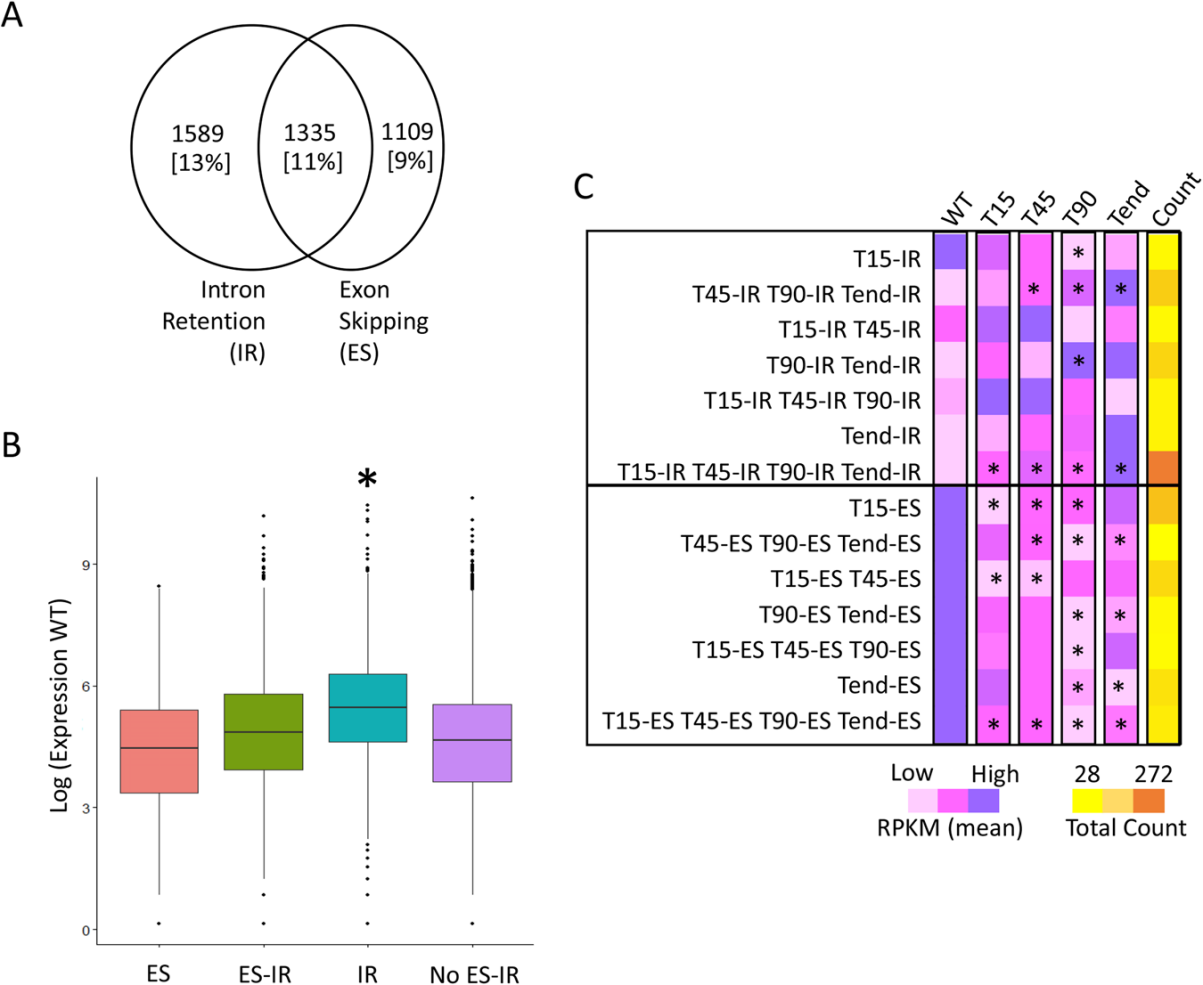
Alternative splicing in *P. tricornutum*. The Venn diagram depicts (**A**) the number of genes predicted to undergo alternative splicing in the context of intron retention (IR) and exon-skipping (ES). Numbers and percentages (in square brackets) in the Venn diagram reflect the absolute number of genes and the relative percentage out of the total Phatr3 genes. (**B**) The box-plot represents the differences in expression of genes corresponding to different functional categories, represented on x-axis. Y-axis represents the expression of the genes in the wild-type (WT) (Biosample accession: SAMN06350643), scaled to log scale. (**C**) The heat-map compares the average expression of genes exhibiting intron-retention/exon-skipping (IR/ES) at different time-points (T15, T45, T90 and Tend) in Nfree culture conditions. For example, T15-IR row compares the average expression of all the genes exhibiting intron retention (IR) are time T15 across all the time-points. The last column represents the heat-map based on the number of genes in each category indicated on left Y-axis. *Indicates the level of significance as being significant (P-value < 0.05, two sample t-test with unequal variance) when the average expression of a particular sub-set is compared to the WT. Nfree in the figure denotes the culture condition with no source of nitrogen, WT denotes wild type or normal condition, T15 denotes RNAseq performed on cells sampled after 15 minutes of the culture, Similarly, T45, T90 and Tend denotes RNAseq libraries were prepared upon sampling the cells after 45 minutes, 90 minutes and 18 hours of culture, respectively.

Next, we profiled alternative splicing events in *P. tricornutum* using RNA-Seq data generated in different growth and stress conditions (see Methods). From the 12,177 Phatr3 gene models, 2,924 (~24%) and 2,444 (~20%) genes undergo IR and ES, respectively. A total of 1,335 (~11%) genes are found to undergo both ES and IR, hence can perform alternative splicing (Fig. 4A; Supplementary Table S1). Like most unicellular eukaryotes and unlike metazoans, *P. tricornutum* shows a higher rate of IR than ES, supporting the hypothesis that ES has become more prevalent over the course of metazoan evolution^39^. We then considered the expression dynamics of *P. tricornutum* genes that undergo IR or ES. Surprisingly, we found that genes that can undergo intron-retention are more highly expressed than genes that do not show alternative splicing (two sample t-test, P-value < 0.008, Fig. 4B). This is in contrast to the situation in mammals in which intron-retention down-regulates the genes that are physiologically less relevant^46^.

To further assess the biological role of alternative splicing, we identified 1341 genes showing IR and 1099 genes with ES during an 18-hour time course under nitrogen-free growth conditions which is a limiting nutrient for life in the oceans (see Methods). By comparing the expression levels and intron dynamics of each gene during the time course, we found a significant increase in the expression levels of genes undergoing intron retention (two sample t-test with unequal variance, P-value < 0.05) (Fig. 4C; Supplementary Table S1). In contrast, we observed a significant decrease in the expression of genes undergoing ES across different time-points of nitrogen-free growth (two sample t-test with unequal variance, P-value < 0.05) (Fig. 4C). This indicates that the role of restructuring of genes via AS is non-trivial in maintaining the physiology of the cells in response to the environment.

We further examined the functions of the genes that are alternatively spliced under nitrogen starvation. We identified 81 GO categories which were significantly over-represented in either IR or ES across all or different time-points and with plausible functions in tolerating nitrogen starvation (Supplementary Table S7; Supplementary Fig. S7). For example, many of the genes that show enhanced intron-retention following 45 minutes of starvation are implicated in cell cycle regulation (e.g., functions in chromosome separation, histone modifications, or the mitotic cell-cycle checkpoint), which might correspond to an arrest of the cell cycle under nitrate limitation^47^. Similarly, genes implicated in catabolism and storage of cellular nitrogen pools and nitrate and nitrite transport show enhanced intron-retention within 45 to 90 minutes of starvation induction^48^. Our study points to the importance of intragenic regulatory elements in the generation of alternative messenger RNAs increasing functional diversity in diatoms, which should be taken into consideration when performing functional studies in these species.

### Copia-type LTRs make up most of the TEs in the *P. tricornutum* genome

We also revisited the annotation of repetitive elements in the Phatr3 genome assembly by applying a robust and *de novo* approach (see Methods). Repeats were collectively found to contribute ~3.4 Mb (12%) of the assembly, including transposable elements (TEs), unclassified and tandem repeats, as well as fragments of host genes (Table 2). TEs represent 75% of the repeat set, i.e., 2.3 Mb as compared to 1.7 Mb in the previous TE annotation. By comparing the Phatr3 repertoire of TEs, including both large and small elements, with the previous TE annotations, 1,988 (~54%) TEs were found to be novel (Supplementary Table S8).

**Table 2:** Composition of repetitive sequence content.

Type	Class	Order	Superfamily	Phatr3	Coverage (bp)	Phatr2	Coverage (bp)
Transposable Elements (TEs)	Class I	LTR retrotransposons	Copia	1,434	2,083,619	1,869	1,735,622
			DIRS	14	18,118	—	—
			Putative TRIM/LARD	27	7,291	—	—
		Non-LTR retrotransposons	Putative SINE	13	1,454	—	—
	Class II	Subclass I	MuDR	152	100,450	197	87,637
			PiggyBac	65	79,036	116	73,479
			Other transposase	9	12,068	—	—
			Putative non-autonomous	261	182,589	—	—
		Subclass II	Crypton	13	16,556	—	—
	Class III		MITE	235	150,035	—	—
	Putative TEs		Confused TE	52	98,167	35	20,289
Others	Undetermined		Unclassified repeats	873	421,894	—	—
	Host genes		Putative host genes	302	255,568	—	—
	SSR		SSR	255	36,721	1,135	58,267
	Low-complexity repeats			—	—	141	3,401
			Total	3,705	3,373,161	3,493	1,834,123

Previous examination of repetitive elements relied mainly on a library of manually curated TEs^16,49^. In the present work, the *de-novo* annotation using current state-of-the-art approaches (see Methods) with all types of repeated elements yields more elements of the main classes of TEs in Phatr3. As a result, we detected more Copia-type elements, which cover approximately 200 kb of the genome, while Gypsy-type LTR-RTs remain undetected. Furthermore, we detected for the first time Miniature inverted–repeat transposable elements (MITE) in a diatom. These elements are known to be prevalent in plants and animals playing a major role in genomes organization and species evolution. SINE elements were also found for the first time in a diatom. Considering their role in mRNA splicing, protein translation and allele expression bias documented in other species such as mammals, plants and some invertebrates^50,51^, one can imagine similar roles in the diversification of diatom genomes. Note that SINE elements were reported to be missing in most unicellular species^51^.

Next, we considered the epigenetic marks and expression profiles associated with TEs within Phatr3. Consistent with previous reports^15,16^, the majority (2790, ~75%) of the Phatr3 TE repertoire is associated with one or other studied chromatin marks known to maintain either active (H3K4me2, H3K9_14Ac) and/or repressive states (DNA methylation, H3K27me3, H3K9me2, H3K9me3) of the genome (Supplementary Fig. S8A; Table S8). In contrast to coding regions (including the new gene models), ~50% (1845) of the TEs are marked solely by repressive marks (Supplementary Fig. S8A; Supplementary Table S8), suggesting the importance of keeping these TEs under tight control, whereas only ~7% (268) TEs are marked solely by active chromatin marks (Supplementary Fig. S8A; Supplementary Table S8). A total of 458 (~23%) TEs were DNA methylated, most of which (368; ~80%) in a CG context only (Supplementary Fig. S8C; Supplementary Table S8) and express at lower levels compared to those specifically methylated in CHG or CHH contexts (Supplementary Fig. S8E).

Finally, we compared the epigenetic marks and expression profiles associated with different types of TEs and noted distinct patterns of DNA methylation and histone modifications associated with class I and II TEs. Class I TEs are enriched with CG methylation, co-localizing with or without CHH methylation, while class II TEs are predominantly marked by CHH DNA methylation (Supplementary Fig. S9). This specific pattern might also be relevant to the nature of replication of each class of TEs (copy and paste versus cut and paste). Class I TE copy number can rapidly increase, and their higher methylation in both CG and CHG contexts might be a mean to keep their expression under tight control. An interesting pattern of co-occurrence of epigenetic marks over TEs emerges from this analysis, which shows a systematic repression of TEs when associated with CHG methylation (Supplementary Fig. S9). Although the presence of CHG methylation is associated with active marks such as H3K9/14 Ac and H3K4me2, both classes of TEs show a decrease in their expression, suggesting the importance of maintenance of a heterochromatic environment. When checked, many of the TEs with CHG methylation were found to be inserted into or overlapping with genes (Table S8), reflecting the importance of maintaining these TEs in a silent state to not interfere with gene expression. A similar phenomenon has been observed for *Arabidopsis* TEs which are inserted into genes and whose repression is required to avoid the deleterious effects of TE insertion into host genes^52^. This suggests the conservation of epigenetic mediated regulation of TEs between diatoms and higher eukaryotes. Dissecting the epigenetic environment of genes in *P. tricornutum* and algae in general is important to better understand their regulation and provide sufficient knowledge for editing the epigenome using for instance CRISPR cas9 or TALEN to answer fundamental questions about the biology and ecology of these organisms or simply manipulate the epigenome for biotechnology purposes.

In summary, the reannotation of the *P. tricornutum* genome reported here has led to the discovery of a significant number of novel genes with an important proportion of Rv genes fused to additional functional domains, suggesting a role for Rv in restructuring genes during diatom evolution. Furthermore, our work provides new insights into the role of alternative splicing which we found to be abundant in *P. tricornutum* and is likely to play a major role along with epigenetics in the diatom response to environmental cues. Finally, our study contributes to better understand the complex chimeric nature of diatom genomes, supporting the occurrence of multiple gene transfers from green and red algal lineages as well as from bacteria. Overall, our data provide insights into the genetic and evolutionary factors that contribute to the ecological dominance and success of diatoms in contemporary oceans.

## Methods

### Gene discovery and annotation

#### Structural re-annotation

The *P. tricornutum* version 2 genome (JGI), published in the year 2008^7^, was comprehensively structurally re-annotated using multiple RNA sequencing libraries, non-redundant ESTs and updated repertoires of stramenopile proteomes. 42 RNA-Seq libraries generated using an Illumina sequencing platform (Bio-sample accession numbers SAMN04488978-SAMN04489007 and SAMN06350641-SAMN06350652) were mapped to the genome using Genomic Short-read Nucleotide Alignment Program, GSNAP^53^, integrated within mapping pipeline of Ensembl Genomes^54^. The remaining 49 SoLiD sequence libraries (SRA: SRP069841) were aligned to the genome in color space^55^. The alignment file for each mapped library can be accessed from ftp://ftp.ensemblgenomes.org/pub/misc_data/bam/protists/phatr3/. The mapping percentage was estimated using Samtools and varied between 70–96% (Table S9). Transcript assembly was further executed using Cufflinks^56^ with default parameters. The resulting unfiltered transcripts along with EST libraries and protein sequences from stramenopile UniProt Reference Clusters (UniRef90)^57^ were then used for the genome re-annotation. The structural units derived were finally used to train SNAP^58^ and Augustus^59^ gene prediction programs using default parameters of the MAKER2 annotation pipeline^60^.

#### Functional re-annotation

All predicted gene models were annotated for protein function using InterProScan^61^ as part of the Ensembl protein features pipeline^62^. The results are available at http://protists.ensembl.org/Phaeodactylum_tricornutum/Info/Index/. We also used CLADE and DAMA to enhance the protein domain predictions (see supplementary methods for details). The presence of organelle signaling signatures within the entire Phatr3 gene repertoire was further investigated using ASAFind and HECTAR, under the default conditions as specified in the original publications for each program^13,63^. HECTAR was run remotely, using the Galaxy integrated server provided by the Roscoff Culture Collection (http://webtools.sb-roscoff.fr/).

#### Distribution of epigenetic marks

Data corresponding to histone H3 post-translation modification marks, H3K27me3; H3K9me2/3; H3K14_Ac; H3K4me2 and DNA methylation (CG, CHH, CHG), were taken from^15^ and^16,64^, respectively. RNA-Seq data for Pt18.6 (normal growth condition) was also downloaded from the same resource. Distribution of all marks along with the expression were then checked over the new genes and new transposable element (TE) models by adapting the methodology applied in^15^ and^16^. Estimation of expression (normalized DESeq counts) and differential gene expression analysis at different stages of the study was performed using Eoulsan^65^, using parameters as indicated in Eoulsan parameter file used, (File S3). Statistical analysis to compare the expression of genes was performed using two-sample t-test with unequal variance.

### Evolution of *P. tricornutum* protein coding genes

#### Construction of a multi-sequence reference dataset

A composite reference library, consisting of 75001602 non-redundant protein sequences was compiled from UniPROT (http://www.uniprot.org/help/uniref) (downloaded February 2016), alongside additional genomic and transcriptomic resources from JGI, MMETSP, and the 1kp project currently not located on UniPROT (http://genome.jgi.doe.gov; http://marinemicroeukaryotes.org/; https://sites.google.com/a/ualberta.ca/onekp/) (Table S4)^18^. To minimize artifacts arising from contamination between different MMETSP libraries, each MMETSP library was first pre-cleaned using a BLAST pipeline as described previously^66^ which identifies a custom similarity threshold between each constituent library above which sequence pairs are inferred to be contaminants. In addition, following the methodology of a previous study^10^, MMETSP libraries from a further twelve species were excluded due to the presence of larger scale systematic contamination (Supplementary Table S4).

The reference sequence library was split into twenty-five prokaryotic sub-categories, including archaea and forty-nine eukaryotic sub-categories, which were finally binned into nine distinct groups^10,21^ (Supplementary Table S4; well described in Supplementary File S2). These are diatoms, non-diatom stramenopiles, non-stramenopile SAR, CCTH (containing cryptomonads and haptophytes), green eukaryotes (including glaucophytes), red algae, amorpheans, prokaryotes and viruses. The taxonomic divisions were designed to reflect both current opinions regarding the global organization of the tree of life, as defined using up-to-date taxonomic information^10,21^, and to provide enhanced resolution of the closest relatives of *P. tricornutum* (i.e., other diatoms, other stramenopiles, and other SAR clade members except for stramenopiles).

### Conservation analysis of P. triconrutum proteome

The evolution of the *P. tricornutum* genome was examined using gene homology searches. Orthologues of each gene were identified from each taxonomic sub-category, following the methodology used in the original *Phaeodactylum* genome annotation, by reciprocal BLAST best hit with an initial threshold e-value of 1 × 10^−10^. To minimize the effects of sequence contamination, and subgroup-specific gene transfer events, genes were only denoted as being shared with a particular group if reciprocal BLAST best in at least two separate taxonomic sub-categories within that group, following methodology established elsewhere^10,67^.

### Reconstruction of probable ancient gene transfer events

A novel pipeline, based on BLAST top hit analysis, was designed to determine the probable gene transfer events that have occurred in the evolution of *P. tricornutum*, based on previous BLAST based reconstructions of gene sharing in other photosynthetic eukaryotes^10,67^. For this analysis, the top BLAST hit from each taxonomic sub-category, for each gene in the *Phaeodactylum* genome, were collated to form a single reference library. To enable the identification of highly divergent or partial copies of each gene, a relaxed threshold e-value (10^−10^), that has previously been used for evolutionary analyses of diatom genomes, was employed^7,11^. Following methodology established in a previous study^10^ and the RbH analysis above, a gene was only deemed to have a particular evolutionary origin if top hits were obtained in two or more sub-categories within a particular lineage, prior to the best hit from another lineage. This analysis was performed for seven different reference libraries, one containing all possible reference sequences (Phatr3), and six deducting relatives of *P. tricornutum* (all pennate diatoms, all diatoms, all ochrophytes, all stramenopiles, all SAR clade members, and all SAR + CCTH clade members). Tabulated outputs for each analysis are provided in Table S3. The results obtained by this pipeline were compared to a subset of 324 Phat3 genes for which single-gene tree topologies generated using the expanded reference library have previously been published^10^, and found to give broadly equivalent results (see Results; Supplementary Fig. S9; Supplementary Table S10).

### Alternative splicing

To explore the set of genes undergoing alternative splicing, exon skipping or intron retention, 17 RNA-Seq samples prepared under different conditions of nutrient availability (Biosample accessions: SAMN06350643, SAMN06350647, SAMN04488978-SAMN04488992) were compared. Only genes that were annotated as having two or more exons, and containing introns with a minimum length of 50 bp, were considered for the analysis. RNA-Seq reads were mapped on the reference genome using Bowtie^68^ with parameters: -n 2 -k 2–best. To filter the significant candidate features, we considered horizontal (along the gene) and vertical coverage (depth of reads) to be more than 80% and 4x, respectively. Theoretical support to the candidate features showing exon-skipping or intron-retention was provided by measuring the rate of consensus observation within multiple samples studied. For exons anticipated to show exon-skipping, the observation had a consensus from more than 20% and less than 80% samples. On the other hand, introns having a consensus observation of their retention from more than 20% samples were considered as true events. Functional association studies were further performed on genes showing evidence for exon-skipping or intron-retention. Genes were clustered based on conditions used to prepare the RNA-Seq samples and gene ontology (GO) terms were assigned to the genes (wherever possible) using UniProt-GOA (http://www.ebi.ac.uk/GOA). Significance of these terms was interpreted by calculating the observed to expected ratio of their percent occurring enrichment. The occurrence of an individual biological process within a specific functional set (genes exhibiting intron-retention/exon-skipping, etc.) was compared to that of its occurrence in the complete annotated Phatr3 biological process catalog. The degree of significance of enrichment of each biological process was quantified using a chi-squared test, with a threshold significance P value of 0.05.

To gain insights into the role of alternate splicing in regulating the molecular physiology of the cells, the expression patterns of AS candidates predicted to undergo IR and/or ES under nitrogen replete conditions (Biosample accessions: SAMN04488981, SAMN04488985, SAMN04488979, SAMN04488989) were compared at different time-points (T15min, T45min, T90min and T18hrs (referred as Tend) to the wild-type (Biosample accession: SAMN06350643). RNA-Seq expression values were calculated using Eoulsan^65^, and additionally normalized to eliminate biases caused by the restructuring of alternatively spliced transcripts.

### Annotation of repetitive elements

The REPET v2.2 package^69^ was used to detect the repetitive fraction of the *P. tricornutum* genome. The TEdenovo pipeline (https://urgi.versailles.inra.fr/Tools /REPET) was launched including the Repeat Scout approach^70^ to build a library of consensus sequences representatives of the repeated elements in the genome assembly. The classification comes from decision rules applied to the evidence collected from the consensus sequences. These include: search for structural features, search for tandem repeats, comparison to PFAM, comparison to Repbase^71^ and to a local library of known TEs. The library of manually curated TEs that has been established in previous work^49^ as appended to the TE denovo library and redundancy was removed from the combined library. The TE annotation pipeline was then run with default settings using the sequences from the filtered combined library as probes.

### Data availability

The dataset generated and analyzed during the current study is publically available on SRA, NCBI database. The accession IDs are mentioned within the manuscript text, whenever appropriate.

## Electronic supplementary material

Supplementary information

File S2

File S5

Table S1

Table S2

Table S3

Table S4

Table S5

Table S6

Table S7

Table S8

Table S9

Table S10

Table S11
