# Supplementary material for: Integrative analysis of large scale transcriptome data draws a comprehensive landscape of Phaeodactylum tricornutum genome and evolutionary origin of diatoms
Source: Sci Rep. 2018 Mar 19;8:4834. doi: 10.1038/s41598-018-23106-x (PMC5859163; doi:10.1038/s41598-018-23106-x)
Supplement: Supplementary file 1 — Supplementary information [file 41598_2018_23106_MOESM1_ESM.pdf]

Integrative analysis of large scale transcriptome data draws a comprehensive landscape of *Phaeodactylum tricornutum* genome and evolutionary origin of diatoms

Achal Rastogi, Uma Maheswari, Richard G. Dorrell, Fabio Rocha Jimenez Vieira, Florian Maumus, Adam Kustka, James McCarthy, Andy E. Allen, Paul Kersey, Chris Bowler and Leila Tirichine

Supplementary Figures

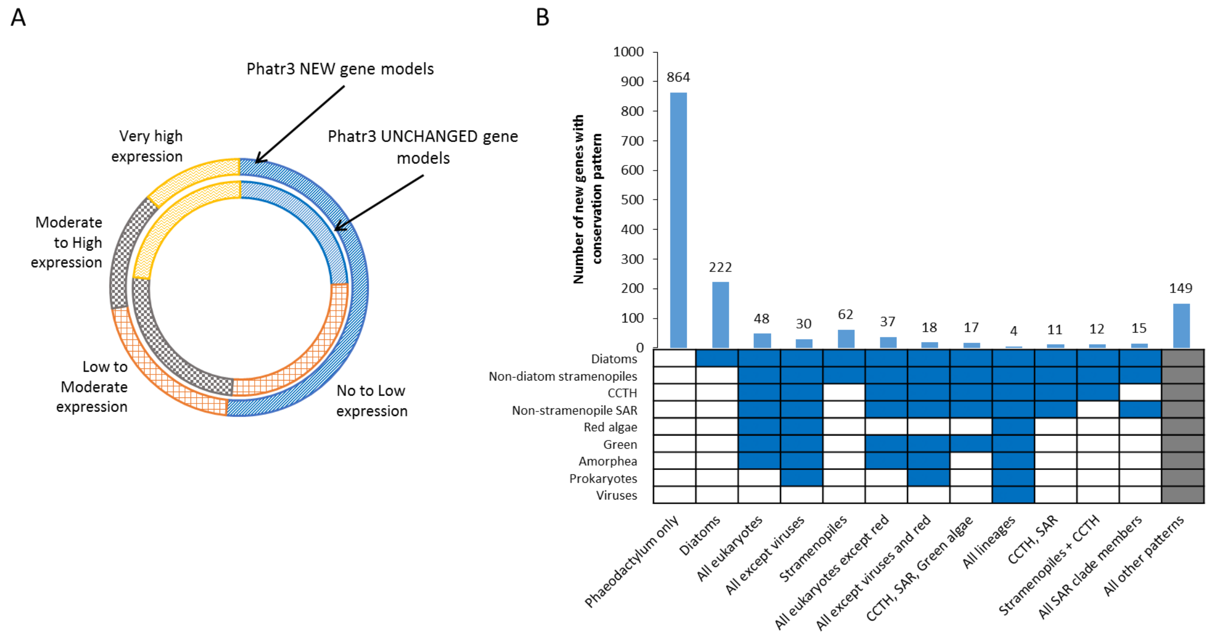

Supplementary Figure 1. Novel genes in Phatr3. (A) The circular stack plot represents the comparison of the expression profile between the proportion of novel Phatr3 gene models (outer circle) with that of the proportion of unchanged Phatr3 gene models (inner circle). (B) The heatmap and graph are shown as per Fig 1.

A

B

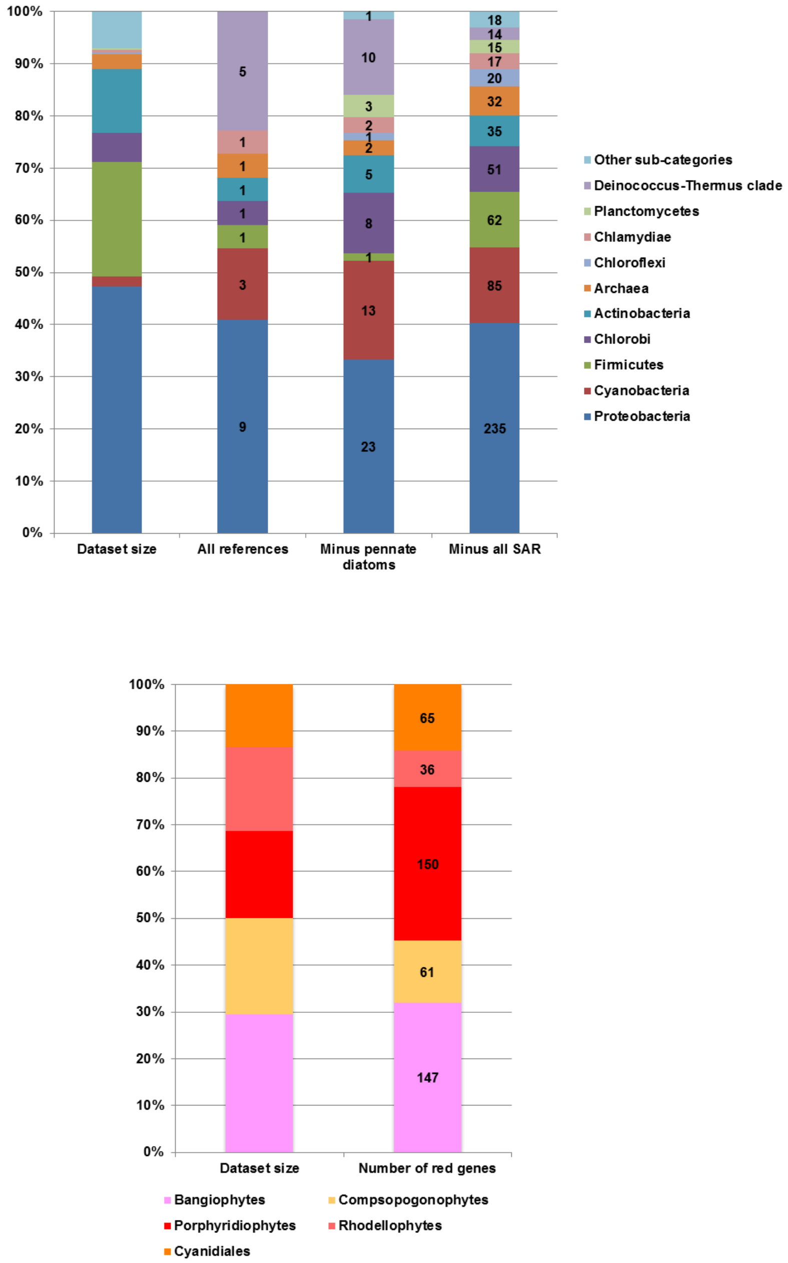

Supplementary Figure 2. Specific taxonomic affiliations of Prokaryotic, Red and Green genes. Each chart shows the specific sub-category from which different prokaryotic (A), and red (B) genes arose. All genes that were assigned (i.e., two or more top hits from two or more sub-categories from a particular lineage, prior to the first top hit from outside that lineage) using the most reduced reference dataset (i.e., all reference sequences, excluding SAR clade members, and other algal lineages with secondary or tertiary plastids) is shown. For prokaryotic genes, two other distributions (obtained for the entire dataset minus non-ochrophyte algae with secondary or tertiary plastids, and the entire dataset minus pennate diatoms, and all non-ochrophyte algae with secondary or tertiary plastids) are shown. Each chart additionally shows the relative size of each sub-category within the reference sequence library, demonstrating that certain sub-categories contribute to substantially more of the top hits (e.g., the *Deinococcus-Thermus* clade in the distribution of prokaryotic genes for the full and pennate diatom-free datasets that were modified to remove all non-ochrophyte lineages with secondary or tertiary plastids) or fewer of the top hits (e.g., the streptophytes, in the distribution of green genes for the dataset from which all SAR clade sequences, and other non-ochrophyte lineages with secondary or tertiary plastids were removed) than might be expected given the corresponding dataset size.

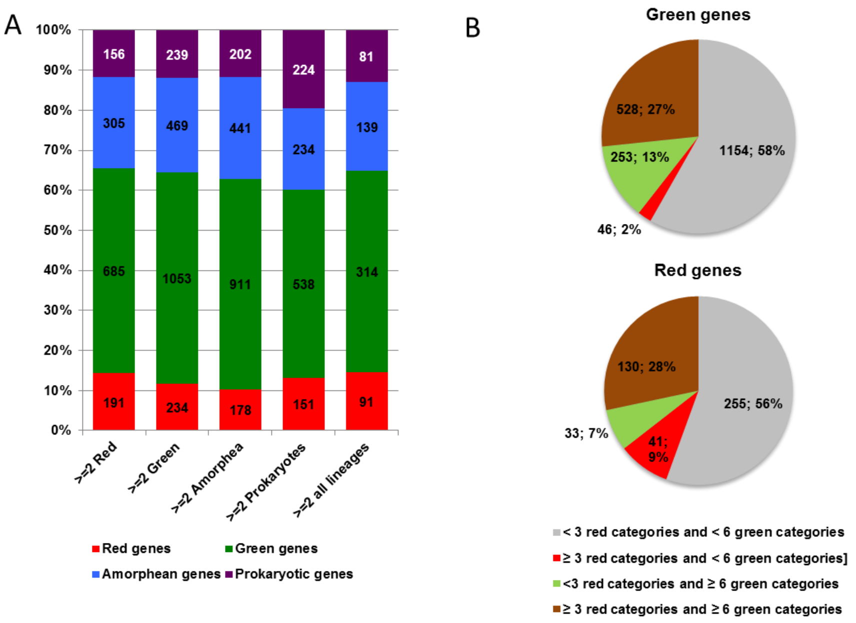

Supplementary Figure 3. Green genes are not purely a result of taxonomic undersampling or lineage-specific gene loss. (A) shows the taxonomic affiliations of genes identified by BLAST top hit analysis, with the dataset from which all SAR clade sequences, and other non-ochrophyte lineages with secondary or tertiary plastids were removed, for which orthologues could be identified in at least two red, green, amorphean or prokaryotic sub-categories, and for which orthologues could be identified in at least two each of the red, green, amorphean and prokaryotic sub-categories. In each case, substantially more genes of green affinity were identified than of other taxonomic affiliation. (B) Compares the number of genes of red or green taxonomic affiliation for which RbH orthologues could be identified in a majority of red (3/5) or green (6/11) sub-categories. A similar proportion of genes of inferred red origin (130/459, 28%) and genes of inferred green origin (528/ 1,981, 27%) were found to have orthologues in a majority of both red and green sub-categories, indicating that the identification of green genes within the dataset was not unfairly biased by taxonomic undersampling of red lineages.

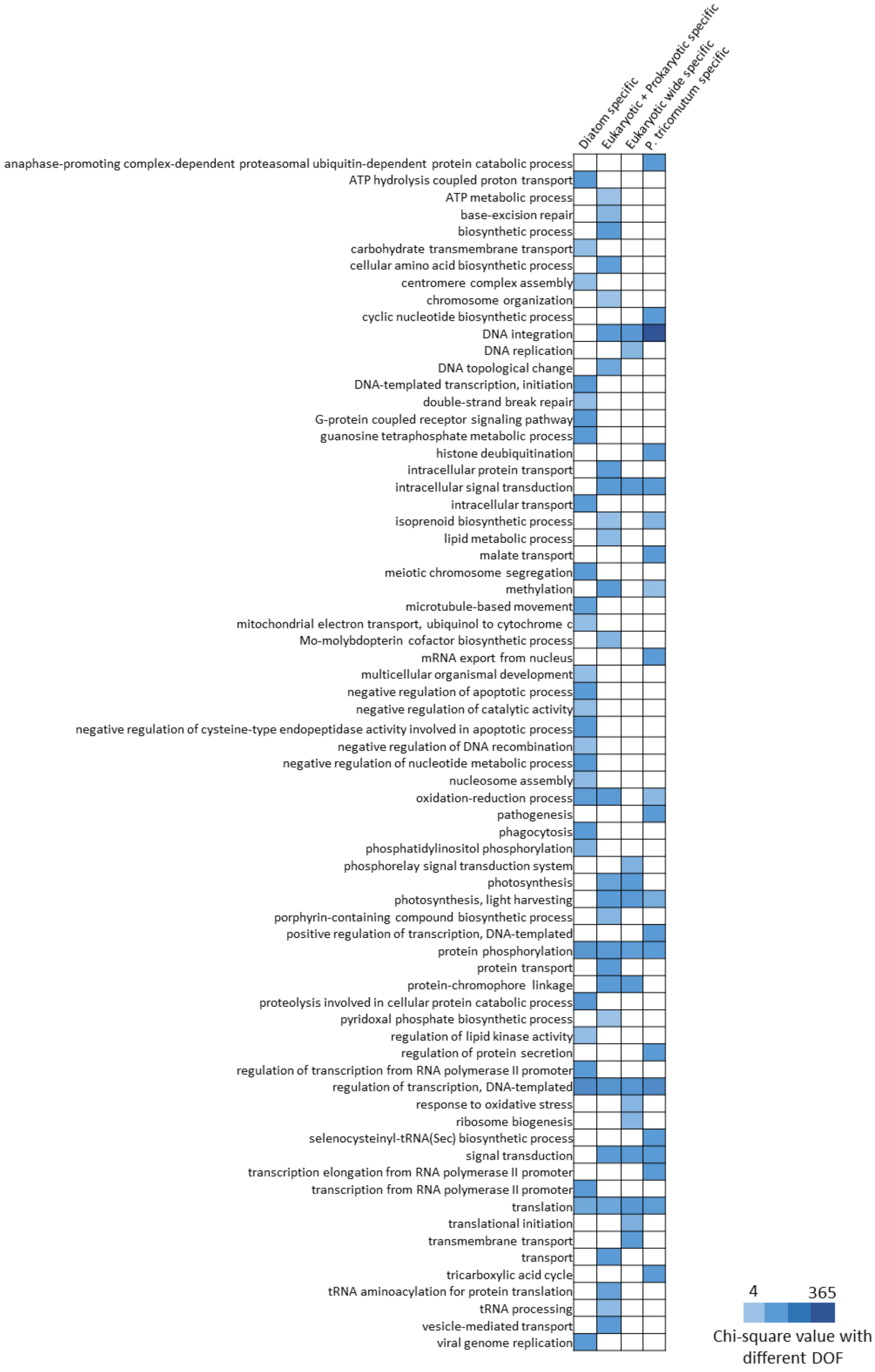

Supplementary Figure 4. Enrichment of biological processes within genes identified to be specific to different groups of organisms. The heat map, indicating chi-square values which are significant (P-value < 0.05) with different degrees of freedom (DOF), depicts various biological processes (left Y-axis) that are enriched in the pool of genes found specific to different groups of organisms (top X-axis). Chi-square values are used to rank the most significant biological processes in descending order. High chi-square value here indicates higher significance (very low P-value) compared to low chi-square values indicating higher P-value but < 0.05.

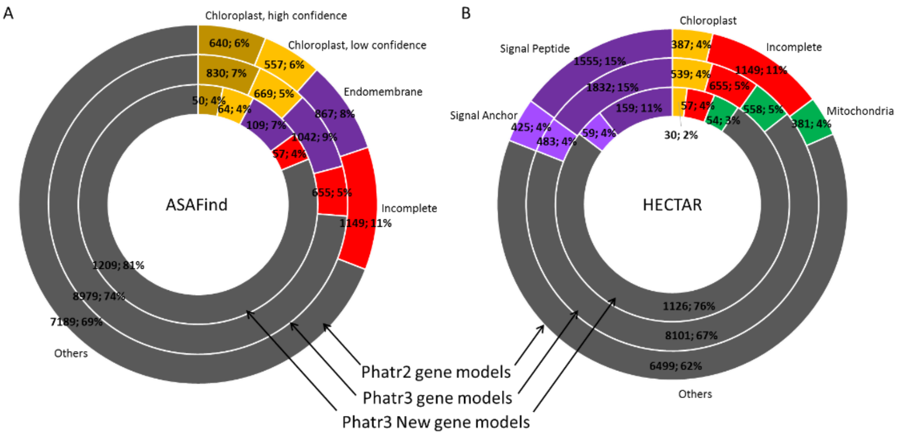

Supplementary Figure 5. Predicted subcellular localization of *P. tricornutum* proteins. This figure shows the targeting predictions for proteins encoded within the *P. tricornutum* genome as assessed using the diatom targeting predictor programmes (A) ASAFind (Gruber et al., 2015) and (B) HECTAR (Gschoessl et al., 2008). The figure is in accordance with Figure 3 panel A and B.

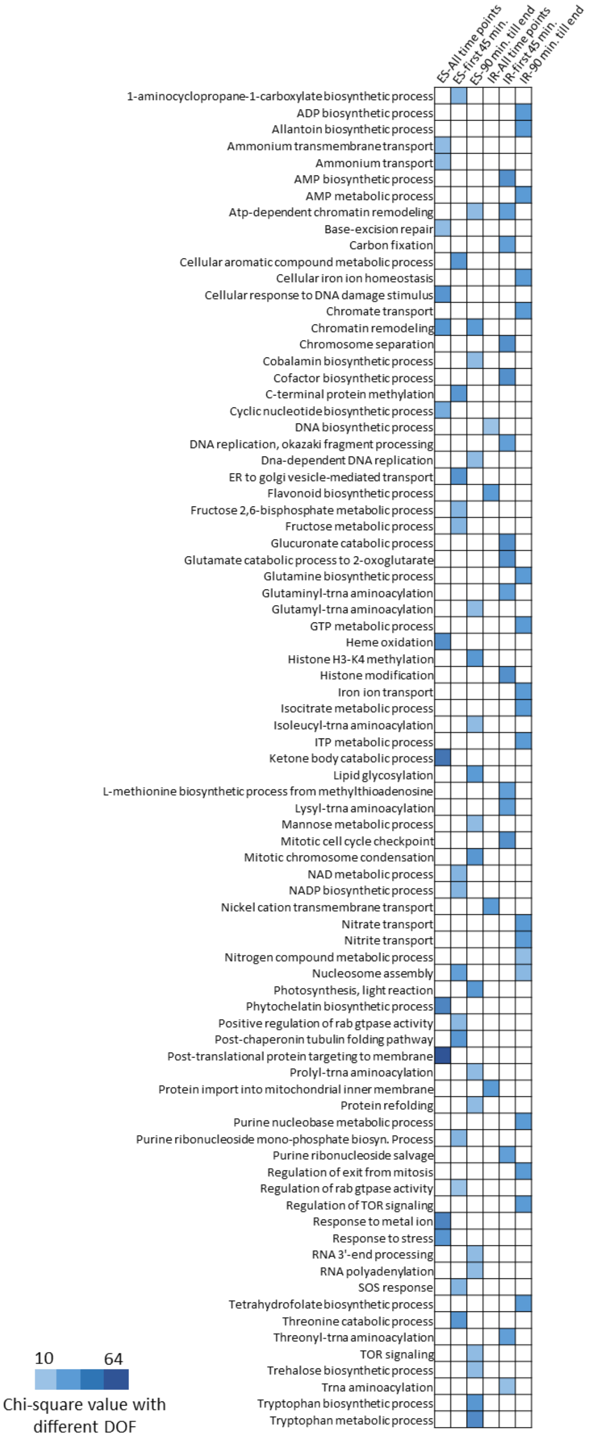

Supplementary Figure 6. Enrichment of biological processes within genes exhibiting alternative splicing at various time-points under Nfree culture conditions. The heat map, indicating chi-square values which are significant (P-value < 0.05) with different degrees of freedom, depicts various biological processes (left Y-axis) that are enriched in the pool of genes exhibiting alternative splicing in the context of intron-retention and exon-skipping (top X-axis). The figure is in relation with categories represented in Figure 4 panel C. Chi-square values are used to rank the most significant biological processes in descending order. High chi-square value here indicates higher significance (very low P-value) compared to low chi-square values indicating higher P-value but < 0.05.

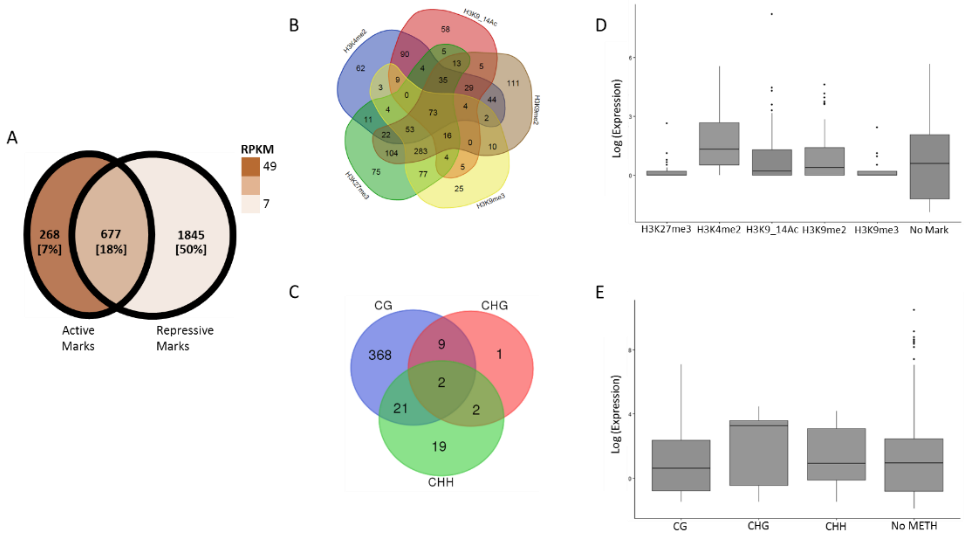

Supplementary Figure 7. Distribution of epigenetic modifications over transposable elements. The Venn diagram (A) represents different chromatin states maintained based on the association of TEs with repressive, active or both repressive and active chromatin modifiers. Numbers and percentages in the Venn diagram reflects the absolute number of TEs and the relative percentage out of the total Phatr3 TEs. The Venn diagram in (B) presents the number of new TEs found to be localized by one or more histone H3 PTMs, and (C) presents the new TEs methylated in different context (CG, CHH, and CHG) of DNA methylation. Boxplots (D) and (E) represents average (median) expression of genes marked exclusively either of the H3 PTMs and are DNA methylated in either of the context, respectively, in normal condition.

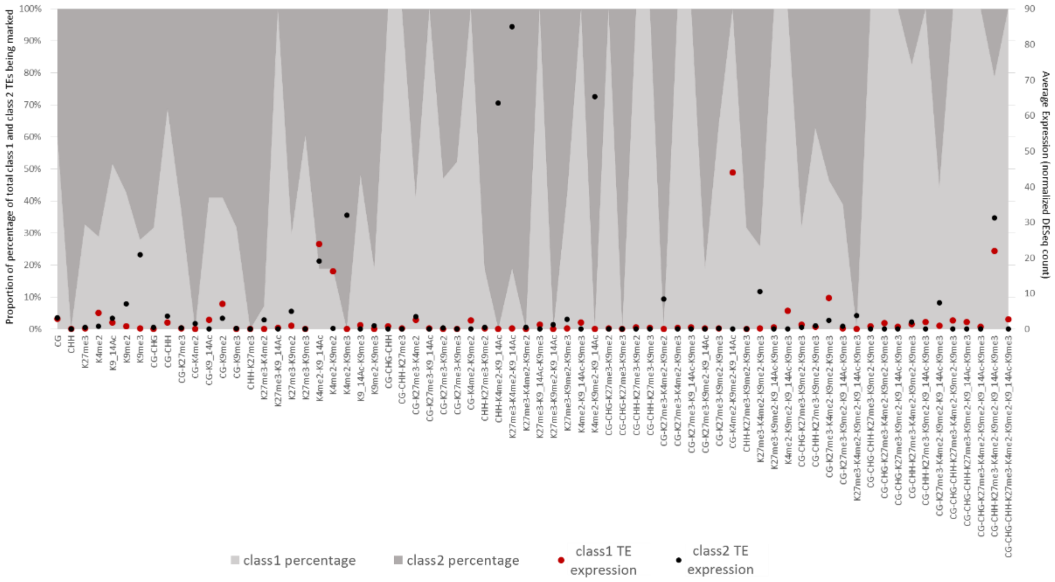

Supplementary Figure 8. Epigenetic marking over transposable elements. The area plot represents the proportion of Class I vs Class II transposable elements being marked by different epigenetic marks including Histone H3 post-translational modifications and DNA methylation (CG, CHH and CHG). Black and red dots indicate the average RNA expression of all the TEs (wherever available) marked in different contexts.

BLAST results obtained using	i) Monophyly of ochrophytes			ii) Sister-group to ochrophytes		
	All data	All except pennate diatoms	All except diatoms	All except ochrophytes	All except stramenopiles	All except SAR
1) BLAST and single-gene tree data comparable						
BLAST and tree topologies congruent	322	314	302	114	109	114
BLAST and tree topologies not congruent	0	0	2	35	35	34
% tree and BLAST topologies congruent	100	100	99.3	76.5	75.7	77.0
2) BLAST and single-gene tree data not comparable						
Not comparable to BLAST- BLAST data insufficiently resolved	2	10	20	141	133	127
Not comparable to tree- topology ambiguous	0	0	0	30	37	39
Not comparable to tree- outgroup sequences not incorporated into tree	0	0	0	4	4	4
Not comparable to tree- sister-group excluded from BLAST analysis	0	0	0	0	6	6

Supplementary Figure 9. Verification of the reconstruction of evolutionary origins by BLAST top hit analysis. (A) Compares the results of BLAST top hit analysis and single-gene phylogeny for 324 genes in Phatr3 incorporated into an independent phylogenetic study of plastid-targeted proteins with broad ochrophyte distribution [10]. Each of the proteins incorporated are found to produce a monophyletic or paraphyletic ochrophyte clade, i.e., should produce BLAST top hits to diatom or other ochrophyte sub-categories in the raw BLAST top hit analysis, and in BLAST top hit analyses from which pennate diatoms and all diatoms have been removed (but other ochrophytes have been retained). In addition, each protein should have a similar BLAST top hit in analyses from which all ochrophyte, stramenopile or SAR clade sequences have been removed to the sister-group to the ochrophyte clade (either red algae, green algae, aplastidic stramenopiles, other eukaryotic lineages, or prokaryotes) inferred from the single-gene tree. The overwhelming majority of the BLAST top hit analyses support monophyly of the ochrophytes, and at least three quarters retrieve the same ochrophyte sister-group as determined through single-gene tree analysis.

Supplementary Table Legends

Supplementary Table 1. The Supplementary File summarizes the findings of the paper in a more holistic view

Supplementary Table 2. Reciprocal best BLAST hit analysis of gene sharing between *Phaeodactylum tricornutum* and other organisms.

Supplementary Table 3. BLAST top hit analysis of gene origin from *Phaeodactylum tricornutum* and multiple reference sequence libraries, excluding non-ochrophyte sub-categories with secondary or tertiary plastids.

Supplementary Table 4. Taxonomic sub-divisions used when constructing the multi-sequence reference library.

Supplementary Table 5. Enriched biological process within genes identified to be specific to different groups of organisms.

Supplementary Table 6. Sub-cellular localization output from ASAFind and HECTAR, analyzed over the entire Phatr3 genes and Phatr3-JGI gene models.

Supplementary Table 7. The file describes all the biological processes which are significantly enriched within genes exhibiting alternative splicing (exon-skipping/intron retention) at different time-points of Nfree culture conditions, compared to all processes Phatr3 annotations.

Supplementary Table 8. File listing the structural and functional annotations of Phatr3 transposable elements (TEs).

Supplementary Table 9. Illumina RNA-Seq libraries used for Phatr3 annotations.

Supplementary Table 10. BLAST top hit analysis of gene origin from *Phaeodactylum tricornutum* and multiple reference sequence libraries.

Supplementary Table 11. Phylip format trimmed alignments, and nexus format tree outputs for each phylogeny

Other Supplementary File Legends

Supplementary File 1. Comparison of certain Phatr3 gene structure with that of Phatr2.

Supplementary File 2. The description of the taxonomic divisions.

Supplementary File 3. Exemplar phylogenetic trees concordant with the BLAST top hit analysis.

This figure presents trees of eight genes within the *P. tricornutum* genome, which encode plastid-targeted proteins with broad evolutionary conservation across the ochrophytes, and a deeper ultimate evolutionary origin involving horizontal transfer from prokaryotes, red or green algae[10]. Trees were calculated from manually curated alignments, using MrBayes and RAxML, under three different substitution matrices (GTR, Jones/JTT, and WAG). The first sheet ("Overview") provides **i)** details of each gene alignment, the tree topology obtained, and compares this to the BLAST output obtained for that gene with the dataset from which all lineages with a suspected history of secondary endosymbiosis were removed, and **ii)** a legend showing the ways in which taxonomic identity and support values are presented in each tree.

The remaining sheets present outputs for each tree. Sheets **A-C** present three trees for genes of ultimate prokaryotic origin: **A** shows an exemplar gene (plastid pyruvate kinase) with limited direct homology outside of the ochrophytes and prokaryotes; and **B-C** show two genes (plastid beta-ketoacyl synthase, and ribulose-5-phosphate 3-epimerase) in which ochrophyte, haptophyte and cryptomonad sequences are more closely related to prokaryotic lineages (respectively chlamydiobacteria and proteobacteria) than other eukaryotes. Sheets **D-E** present two exemplar genes (dual plastid-mitochondrial prolyl tRNA-synthetase, and a periplastid-targeted Mpv17 protein) with deeper red algal affinity. In each case, the ochrophyte, haptophyte and cryptomonad sequences resolve with red algae to the exclusion of aplastidic members of the SAR and CCTH clades (e.g. oomycetes), consistent with a late acquisition of the ochrophyte plastid. Sheets **F-H** present two exemplar genes (plastid DAHP synthetase, and glutathione S-transferase) for which ochrophyte, haptophyte and cryptomonad sequences resolve at specific points within the chlorophyte lineages (respectively Dolichomastigales, and members of the mamiellophytes), and one tree (plastid cyloeucanol cycloisomerase) that posits a deep origin for the ochrophyte sequences within the chlorophyte algae. The presence of red algal orthologues in each tree confirms these genes are not misidentified as a result of undersampling of red lineages, and the evolutionary relationship of these genes to chlorophytes, to the exclusion of streptophyte lineages, indicates a probable horizontal transfer event, as opposed to shared inheritance and differential loss.

Supplementary File 4. EOULSAN parameter Supplementary File specifying the parameters used to analyze the expression using RNA sequencing libraries.

Full list of conserved plastid-targeted proteins identified de novo by Phat3. This lists all of the proteins that have been demonstrated in a separate study (Dorrell et al., manuscript submitted) to form conserved components of the ochrophyte plastid proteome, for which the gene model in Phatr3 encodes a predicted plastid-targeted protein (as inferred either with HECTAR or with ASAFind), and the gene model in Phatr2 is N-incomplete. In each case, the protein cluster identifier (as supplied in Dorrell et al.), functional annotation, and gene ID of each protein in Phat2 and Phat3 are provided.

Identifier	Function	Phat2	Phat3
**2gh**	SAM (and some other nucleotide) binding motif	Phatr2_4846	Phatr3_J4846
**2jh**	Fatty acid desaturase 4	Phatr2_5271	Phatr3_J5271
**2kr**	Heme oxygenase	Phatr2_5851	Phatr3_J5851
**2ks**	Heme oxygenase	Phatr2_5902	Phatr3_J5902
**2kw**	CDP-alcohol phosphatidyltransferase/ Phosphatidylglycerol-phosphate synthase	Phatr2_8663	Phatr3_J8663
**2kx**	Lycopene beta cyclase	Phatr2_8835	Phatr3_J8835
**2la**	Predicted unusual protein kinase	Phatr2_bd116	Phatr3_draftJ116
**2lb**	ATP-dependent Clp protease subunit	Phatr2_7525	Phatr3_draftJ263
**2lc**	CDGSH-type Zn-finger containing protein	Phatr2_bd297	Phatr3_draftJ297
**2lt**	Ferredoxin rieske component	Phatr2_9046	Phatr3_EG02174
**2lv**	Nitrite reductase	Phatr2_13154	Phatr3_EG02286
**2lw**	Ycf49-like protein	Phatr2_11197	Phatr3_EG02357
**aav**	Hypothetical protein	Phatr2_8324	Phatr3_J8324
**xge**	Retinol dehydrogenase	Phatr2_10567	Phatr3_J10567
**xgj**	Predicted unusual protein kinase	Phatr2_12121	Phatr3_J12121
**xgn**	Peroxisomal membrane protein MPV17 and related proteins	Phatr2_12379	Phatr3_J12379
**xgo**	FKBP-type peptidyl-prolyl cis-trans isomerase	Phatr2_12411	Phatr3_J12411
**xgq**	Alkyl hydroperoxide reductase, thiol specific antioxidant and related enzymes	Phatr2_12713	Phatr3_J12713
**xgt**	Uncharacterized conserved protein	Phatr2_13158	Phatr3_J13158
**xgx**	Serine O-acetyltransferase	Phatr2_13476	Phatr3_J13476
**xhb**	Peptide methionine sulfoxide reductase	Phatr2_14769	Phatr3_J14769
**xhc**	Predicted ATPase	Phatr2_1494	Phatr3_J1494
**xhf**	Seryl-tRNA synthetase	Phatr2_15374	Phatr3_J15374
**xhg**	Tryptophanyl-tRNA synthetase	Phatr2_15595	Phatr3_J15595
**xhh**	Glutathione S-transferase	Phatr2_15764	Phatr3_J15764
**xhi**	SsrA-binding protein	Phatr2_16021	Phatr3_J16021
**xhn**	tRNA uracil-5-methyltransferase and related tRNA-modifying enzymes	Phatr2_16911	Phatr3_J16911
**xho**	Glutaminyl-tRNA ligase	Phatr2_16963	Phatr3_J16963
**xhp**	RNA polymerase sigma factor	Phatr2_17029	Phatr3_J17029
**xmv**	Peptidase S41	Phatr2_3061	Phatr3_J3061

Comparative alignments of Phat2 and Phat3 conserved plastid-targeted proteins. This figure shows exemplar alignments of six proteins that are targeted to the plastids of a wide range of photosynthetic stramenopiles, for which the Phat2 gene model is incomplete at the N-terminus. For each protein, a global alignment of all stramenopile plastid-targeted sequences identified is shown (**i**), alongside close-up regions covering the N-termini (**ii**) of the Phat3 (**iia**) and Phat2 (**iib**) gene models. In each case the N-terminus identified by Phat3 broadly matches the N-terminus identified in orthologues from other stramenopile lineages, whereas the N-terminus identified by Phat2 is positioned within the conserved region of the protein. Otherwise conserved regions of sequence that are missing in Phat2 and completed by Phat3 are highlighted using coloured bars.

A) Mv22-type peroxisomal membrane protein

i) Full alignment

ii) N-terminus only

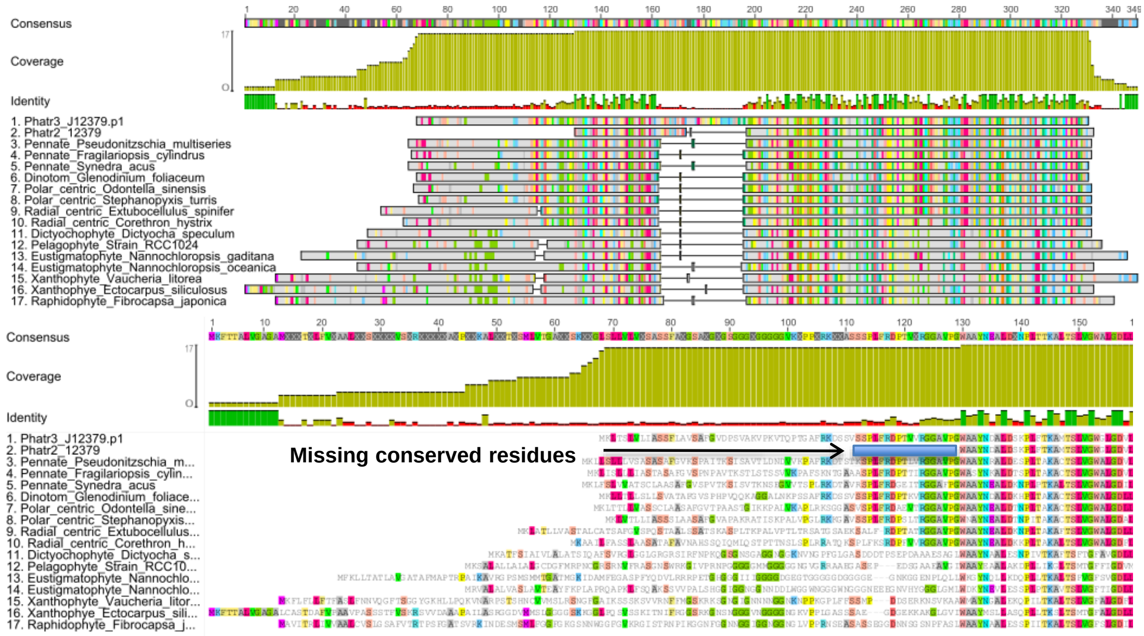

B) S-adenosyl methionine binding protein

i) Full alignment

iia) Phat3 N-terminus

iib) Phat2 N-terminus

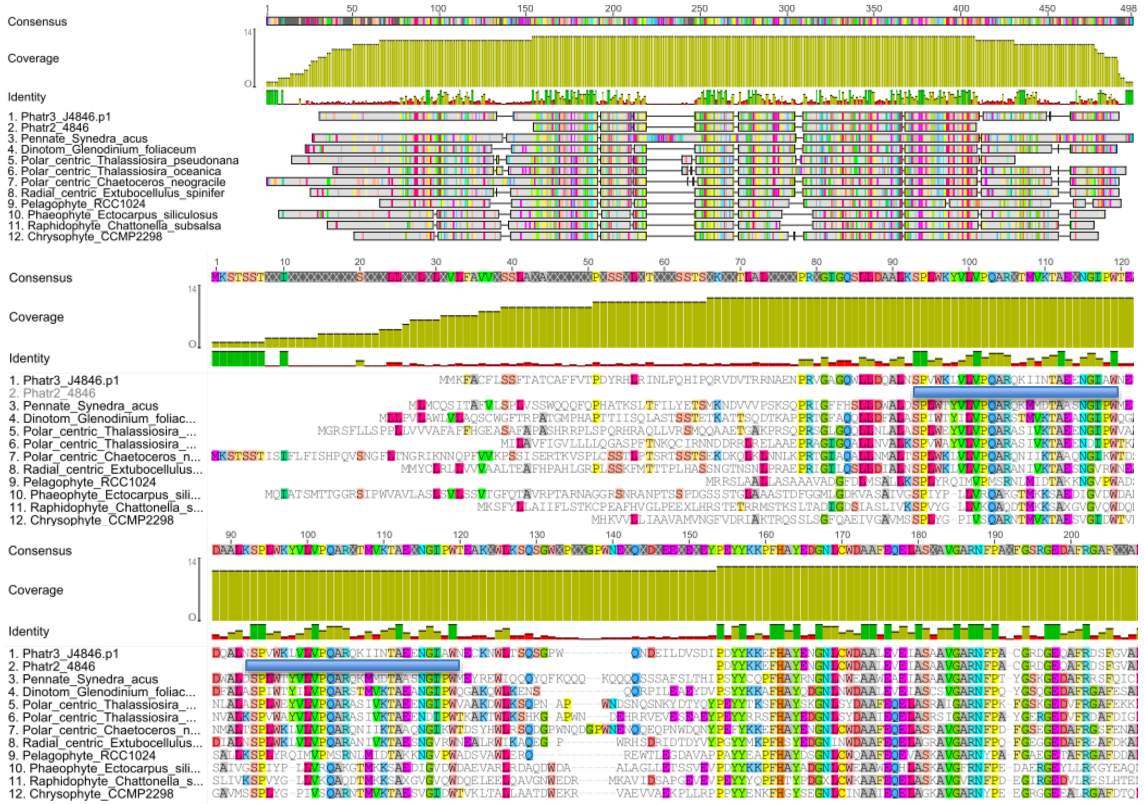

C) Phosphatidyl-glycerolphosphate synthase

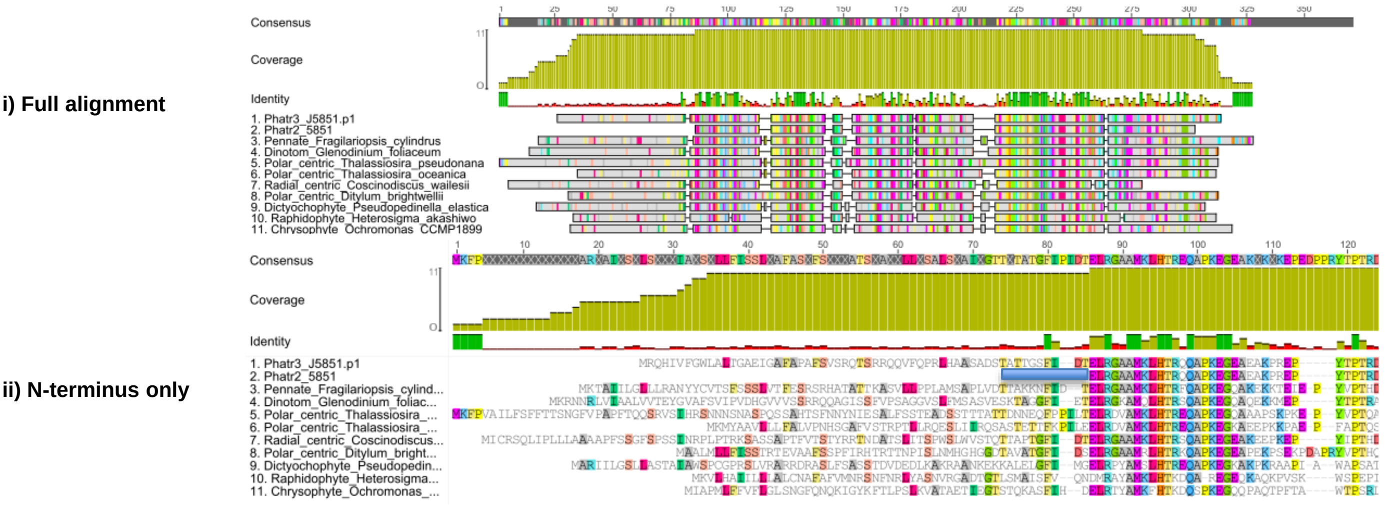

D) Peptidyl-prolyl cis-trans isomerase

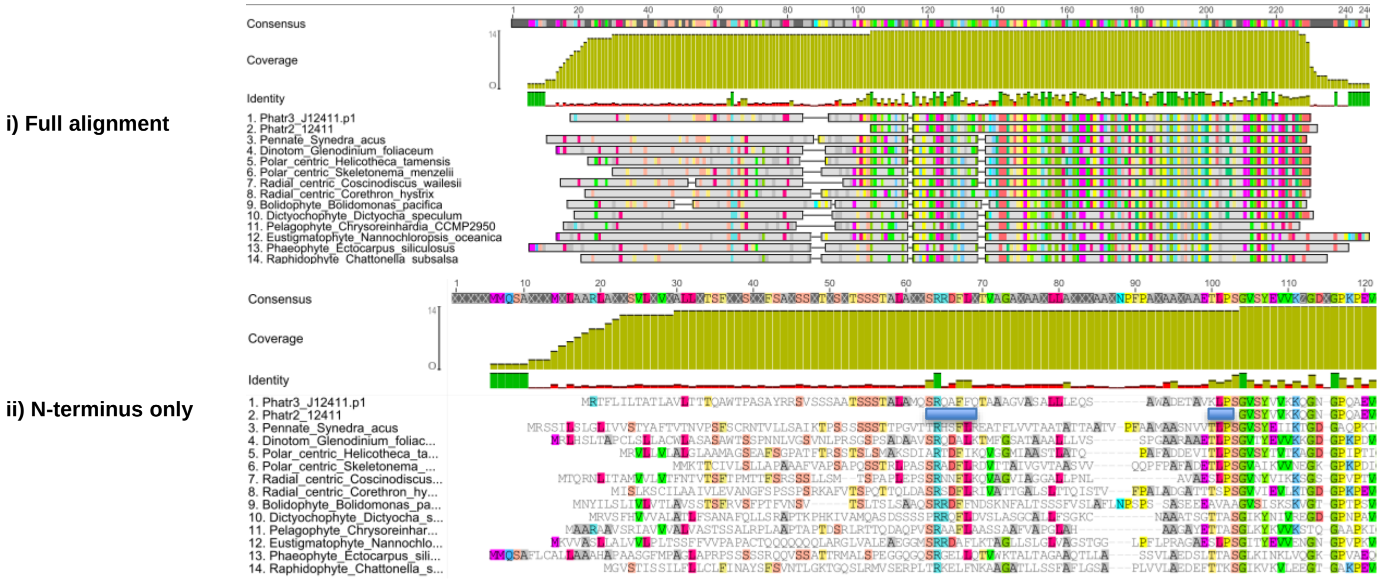

E) Serine O-acetyltransferase

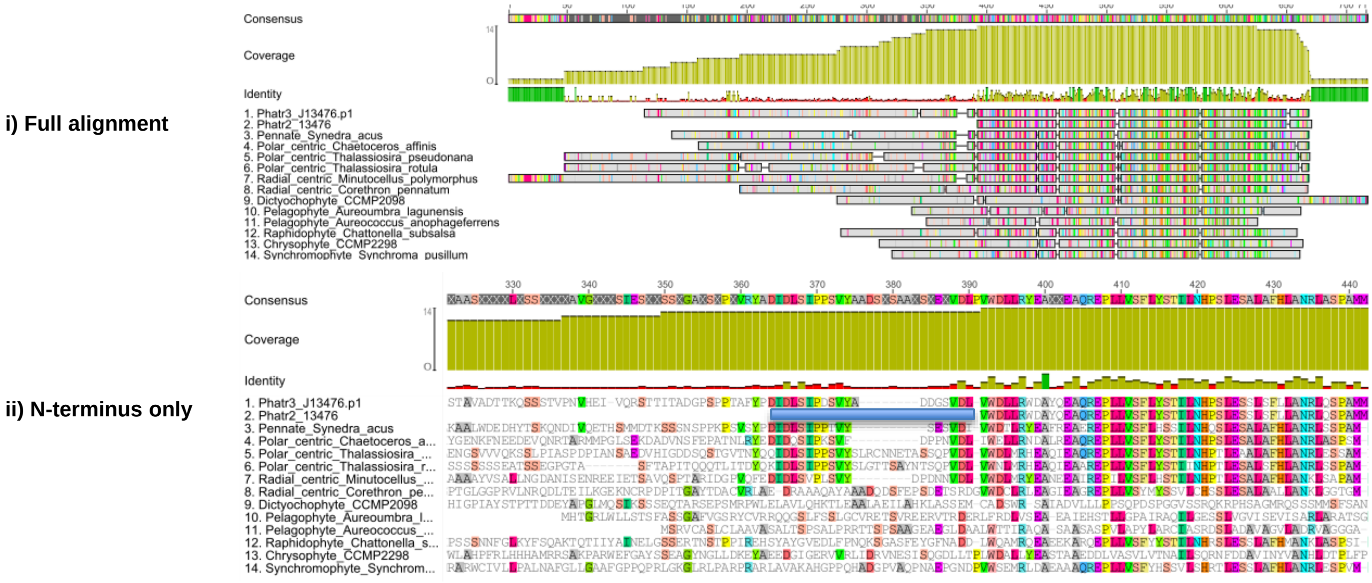

F) Ycf49-like protein

i) Full alignment

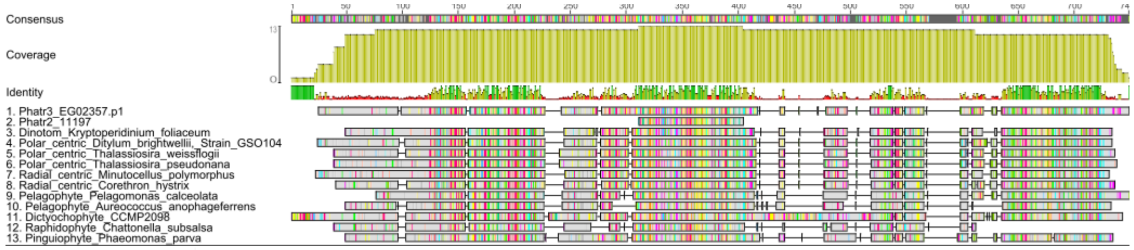

iia) Phat3 N-terminus

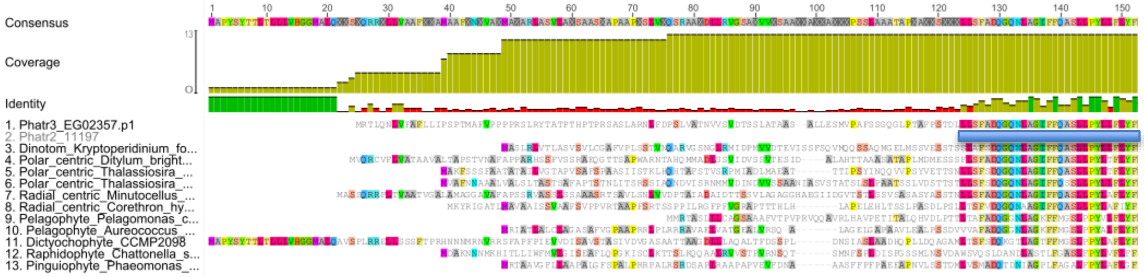

iib) Phat2 N-terminus

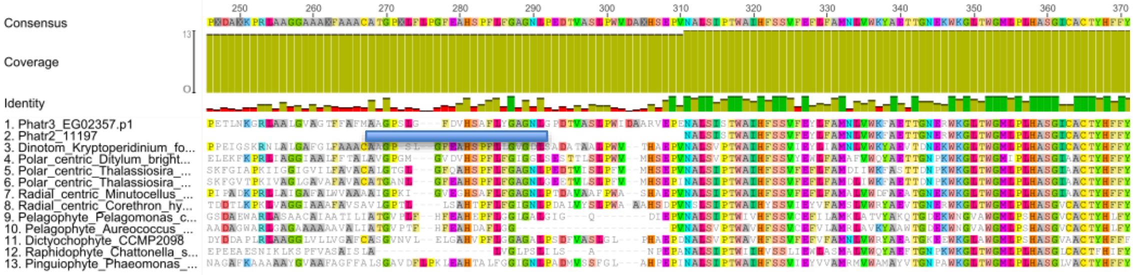

Exemplar RT-PCR of Phat3 conserved plastid-targeted proteins. Panel A tabulates the primers used for RT-PCR amplification of the five conserved plastid-targeted proteins identified specifically by Phat3 (Fig. SB), excluding the previously tested peroxisomal membrane-type protein. **Panel B** shows an exemplar gel photo, showing RT-PCR and cDNA negative control products for each gel. In each case the RT-PCR product gives a band of the expected size, whereas the cDNA negative control product does not. The molecular size standard used is GeneRuler 1 kb ladder (Thermo).

A)

Construct	Gene	PCR F	PCR R	mRNA length (nt)
**1**	S-adenosyl methionine binding protein	ATGATGAAATTTGCCTGTTTTC	TTTTTTTTTATTTTCGAACAC	1206
**2**	Phosphatidyl-glycerolphosphate synthase	ATGACAGTGAATCGCCTTTTTC	CTTACTGTTTGCTTTGAGTAG	897
**3**	Peptidyl-prolyl cis-trans isomerase	ATGCGTACCTTTCTGATTC	ATCATCACCAATGAGTTCAATG	615
**4**	Serine O-acetyltransferase	ATGGTAGCGTGGCTCGTCCTGC	TATCCCGTCCGATTCAAACGTC	1620
**5**	Ycf49-like protein	ATGCGGACTTTGCAGAATCTG	CAGCTTTTTGACGGGTATAAC	1773

B)

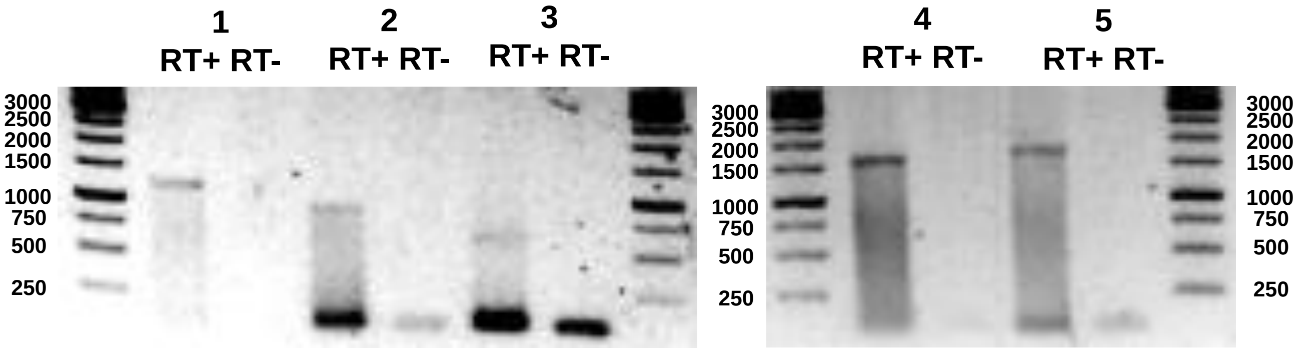

Tree overview

i)

Panel	GeneID	Function	Matrix size (taxa x aa)	Tree topology	BLAST output (excluding other complex algal groups)
A	J22404	Plastid pyruvate kinase	55 x 421	Monophyletic group of ochrophytes and cryptomonads; sister-group to prokaryotes (eluisimicrobia)	Vertically inherited in ochrophytes; previously laterally acquired from prokaryotes (proteobacteria)
B	J37367	Plastid beta-ketoacyl synthase	76 x 405	Monophyletic group of ochrophytes, haptophytes and cryptomonads; sister-group to prokaryotes (chlamydiobacteria)	Vertically inherited in ochrophytes; previously laterally acquired from prokaryotes (chlamydiobacteria)
C	J53935	Plastid D-ribulose-5-phosphate 3-epimerase	70 x 223	Monophyletic group of ochrophytes and cryptomonads; sister-group to prokaryotes (proteobacteria)	Vertically inherited in ochrophytes; previously laterally acquired from prokaryotes (proteobacteria)
D	J43097	Plastid/ mitochondrial prolyl-tRNA synthetase	99 x 493	Monophyletic group of ochrophytes, haptophytes, cryptomonads and red algae	Vertically inherited in ochrophytes; previously laterally acquired from red algae (porphyridiophytes)
E	J12379	Periplastid-targeted Mpv17 protein	104 x 185	Monophyletic group of ochrophytes, haptophytes and red algae	Vertically inherited in ochrophytes; previously laterally acquired from red algae (bangiophytes)
F	J24353	Plastid DAHP synthetase, class II	66 x 492	Monophyletic group of ochrophytes; sister-group to green algae (Dolichomastigales)	Vertically inherited in ochrophytes; previously laterally acquired green algae (streptophytes)
G	J45400	Plastid glutathione S-transferase	50 x 339	Paraphyletic group of ochrophytes, haptophytes and green algae (Pyramimonadales/ Mamiellophytes)	Vertically inherited in ochrophytes; previously laterally acquired green algae (Pyramimonadales)
H	J49447	Plastid cycloeucanol cycloisomerase	55 x 292	Monophyletic group of ochrophytes, haptophytes and cryptomonads; sister-group to green algae (prasinophytes)	Vertically inherited in ochrophytes; previously laterally acquired green algae (Dolichomastigales)

ii)

Key- node support values

- ● Posterior probabilities 1.0 in 3x MrBayes consensus trees (GTR, Jones, WAG)
- ● Posterior probabilities > 0.8 in all MrBayes trees

x/y/z MrBayes: GTR, Jones, WAG
a/b/c RAxML: GTR, JTT, WAG (used for all remaining nodes)

Key- taxon labels

- ● Diatoms
- ● Other stramenopiles
- ● Other SAR
- ● CCTH
- ● Red algae
- ● Green lineages and glaucophytes
- ● Amorphea
- ● Prokaryotes
- ● Viruses

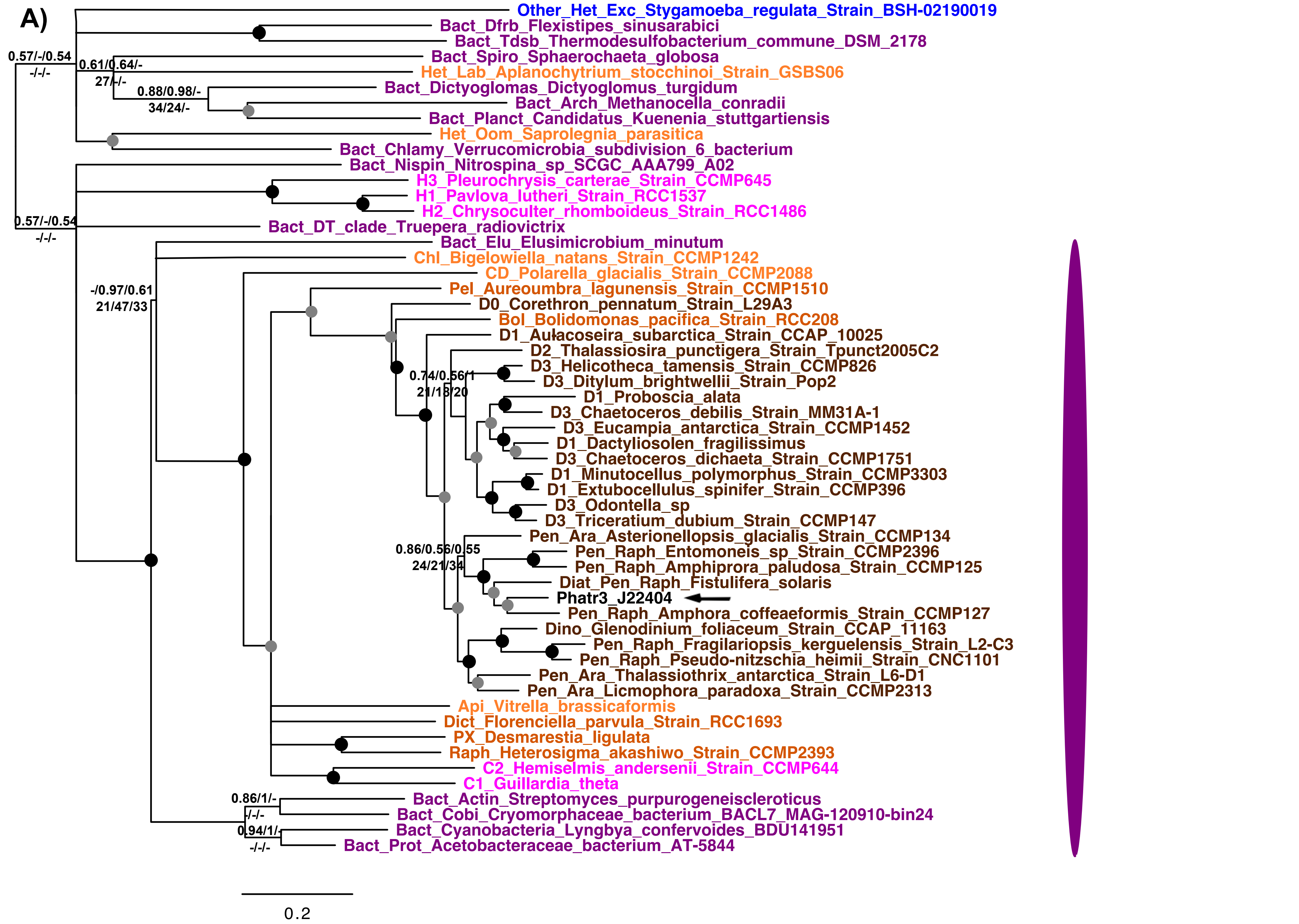
A)
Other_Het_Exc_Stygamoeba_regulata_Strain_BSH-02190019
Bact_Dfrb_Flexistipes_sinusarabici
Bact_Tdsb_Thermodesulfobacterium_commune_DSM_2178
Bact_Spiro_Sphaerochaeta_globosa
Het_Lab_Aplanochytrium_stocchinoi_Strain_GSBS06
Bact_Dictyoglomas_Dictyoglomus_turgidum
Bact_Arch_Methanocella_conradii
Bact_Planct_Candidatus_Kuenenia_stuttgartiensis
Het_Oom_Saprolegnia_parasitica
Bact_Chlamy_Verrucomicrobia_subdivision_6_bacterium
Bact_Nispin_Nitrospina_sp_SCGC_AAA799_A02
H3_Pleurochrysis_carterae_Strain_CCMP645
H1_Pavlova_lutheri_Strain_RCC1537
H2_Chrysoculter_rhomboideus_Strain_RCC1486
Bact_DT_clade_Truepera_radiovictrix
Bact_Elu_Elusimicrobium_minutum
Chl_Bigelowiella_natans_Strain_CCMP1242
CD_Polarella_glacialis_Strain_CCMP2088
Pel_Aureoumbra_lagunensis_Strain_CCMP1510
D0_Corethron_pennatum_Strain_L29A3
Bol_Bolidomonas_pacifica_Strain_RCC208
D1_Aulacoseira_subarctica_Strain_CCAP_10025
D2_Thalassiosira_punctigera_Strain_Tpunct2005C2
D3_Helicotheca_tamensis_Strain_CCMP826
D3_Ditylum_brightwellii_Strain_Pop2
D1_Proboscia_alata
D3_Chaetoceros_debilis_Strain_MM31A-1
D3_Eucampia_antarctica_Strain_CCMP1452
D1_Dactyliosolen_fragilissimus
D3_Chaetoceros_dichaeta_Strain_CCMP1751
D1_Minutocellus_polymorphus_Strain_CCMP3303
D1_Extubocellulus_spinifer_Strain_CCMP396
D3_Odontella_sp
D3_Triceratium_dubium_Strain_CCMP147
Pen_Ara_Asterionellopsis_glacialis_Strain_CCMP134
Pen_Raph_Entomoneis_sp_Strain_CCMP2396
Pen_Raph_Amphiprora_paludosa_Strain_CCMP125
Diat_Pen_Raph_Fistulifera_solaris
Phatr3_J22404
Pen_Raph_Amphora_coffeaeformis_Strain_CCMP127
Dino_Glenodinium_foliaceum_Strain_CCAP_11163
Pen_Raph_Fragilariopsis_kerguelensis_Strain_L2-C3
Pen_Raph_Pseudo-nitzschia_heimii_Strain_CNC1101
Pen_Ara_Thalassiothrix_antarctica_Strain_L6-D1
Pen_Ara_Licmophora_paradoxa_Strain_CCMP2313
Api_Vitrella_brassicaformis
Dict_Florenciella_parvula_Strain_RCC1693
PX_Desmarestia_ligulata
Raph_Heterosigma_akashiwo_Strain_CCMP2393
C2_Hemiselmis_andersenii_Strain_CCMP644
C1_Guillardia_theta
Bact_Actin_Streptomyces_purpurogeneiscleroticus
Bact_Cobi_Cryomorphaceae_bacterium_BACL7_MAG-120910-bin24
Bact_Cyanobacteria_Lyngbya_confervoides_BDU141951
Bact_Prot_Acetobacteraceae_bacterium_AT-5844
0.57/-/0.54
-/-/-
0.61/0.64/-
27/-/-
0.88/0.98/-
34/24/-
0.57/-/0.54
-/-/-
-/0.97/0.61
21/47/33
0.74/0.56/1
21/18/20
0.86/0.56/0.55
24/21/34
0.86/1/-
-/-/-
0.94/1/-
-/-/-
0.2

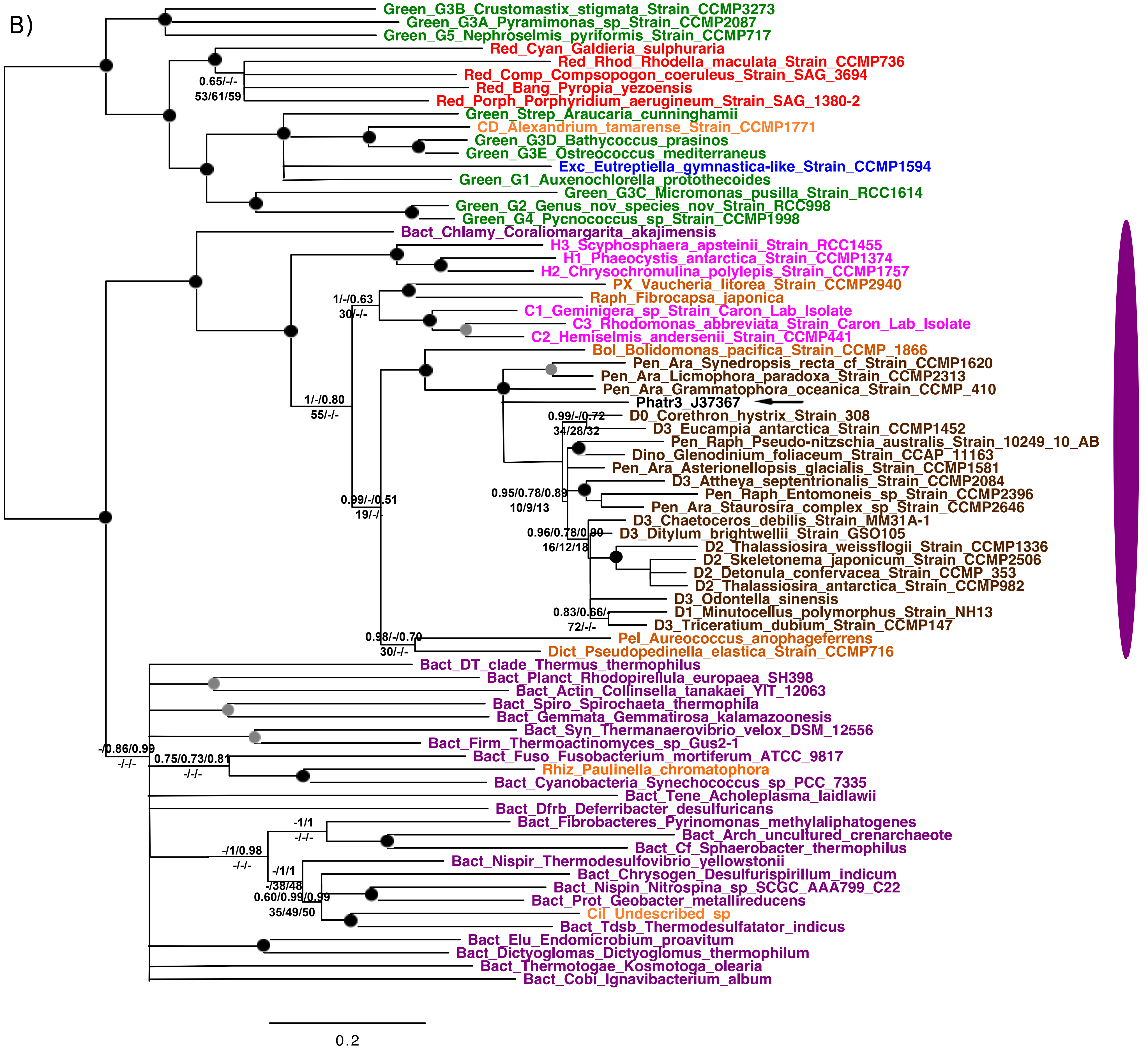

B)
Green_G3B_Crustomastix_stigmata_Strain_CCMP3273
Green_G3A_Pyramimonas_sp_Strain_CCMP2087
Green_G5_Nephroselmis_pyriformis_Strain_CCMP717
Red_Cyan_Galdieria_sulphuraria
Red_Rhod_Rhodella_maculata_Strain_CCMP736
Red_Comp_Compsopogon_coeruleus_Strain_SAG_3694
Red_Bang_Pyropia_yezoensis
Red_Porph_Porphyridium_aerugineum_Strain_SAG_1380-2
0.65/-/-
53/61/59
Green_Strep_Araucaria_cunninghamii
CD_Alexandrium_tamarense_Strain_CCMP1771
Green_G3D_Bathycoccus_prasinos
Green_G3E_Ostreococcus_mediterraneus
Exc_Eutreptiella_gymnastica-like_Strain_CCMP1594
Green_G1_Auxenochlorella_protothecoides
Green_G3C_Micromonas_pusilla_Strain_RCC1614
Green_G2_Genus_nov_species_nov_Strain_RCC998
Green_G4_Pycnococcus_sp_Strain_CCMP1998
Bact_Chlamy_Coraliomargarita_akajimensis
H3_Scyphosphaera_apsteinii_Strain_RCC1455
H1_Phaeocystis_antarctica_Strain_CCMP1374
H2_Chrysochromulina_polylepis_Strain_CCMP1757
PX_Vaucheria_litorea_Strain_CCMP2940
Raph_Fibrocapsa_japonica
1/-/0.63
30/-/-
C1_Geminigera_sp_Strain_Caron_Lab_Isolate
C3_Rhodomonas_abbreviata_Strain_Caron_Lab_Isolate
C2_Hemiselmis_andersenii_Strain_CCMP441
Bol_Bolidomonas_pacifica_Strain_CCMP_1866
Pen_Ara_Synedropsis_recta_cf_Strain_CCMP1620
Pen_Ara_Licmophora_paradoxa_Strain_CCMP2313
Pen_Ara_Grammatophora_oceanica_Strain_CCMP_410
1/-/0.80
55/-/-
Phatr3_J37367
0.99/-/0.72
34/28/32
D0_Corethron_hystrix_Strain_308
D3_Eucampia_antarctica_Strain_CCMP1452
Pen_Raph_Pseudo-nitzschia_australis_Strain_10249_10_AB
Dino_Glenodinium_foliaceum_Strain_CCAP_11163
Pen_Ara_Asterionellopsis_glacialis_Strain_CCMP1581
D3_Attheya_septentrionalis_Strain_CCMP2084
0.95/0.78/0.89
10/9/13
Pen_Raph_Entomoneis_sp_Strain_CCMP2396
Pen_Ara_Staurosira_complex_sp_Strain_CCMP2646
0.99/-/0.51
19/-/-
D3_Chaetoceros_debilis_Strain_MM31A-1
0.96/0.78/0.90
16/12/18
D3_Ditylum_brightwellii_Strain_GSO105
D2_Thalassiosira_weissflogii_Strain_CCMP1336
D2_Skeletonema_japonicum_Strain_CCMP2506
D2_Detonula_confervacea_Strain_CCMP_353
D2_Thalassiosira_antarctica_Strain_CCMP982
D3_Odontella_sinensis
0.83/0.66/
72/-/-
D1_Minutocellus_polymorphus_Strain_NH13
D3_Triceratium_dubium_Strain_CCMP147
0.98/-/0.70
30/-/-
Pel_Aureococcus_anophageferrens
Dict_Pseudopedinella_elastica_Strain_CCMP716
Bact_DT_clade_Thermus_thermophilus
Bact_Planct_Rhodopirellula_europaea_SH398
Bact_Actin_Collinsella_tanakaei_YIT_12063
Bact_Spiro_Spirochaeta_thermophila
Bact_Gemmata_Gemmatirosa_kalamazoonesis
Bact_Syn_Thermanaerovibrio_velox_DSM_12556
Bact_Firm_Thermoactinomyces_sp_Gus2-1
-/0.86/0.99
-/-/-
0.75/0.73/0.81
-/-/-
Bact_Fuso_Fusobacterium_mortiferum_ATCC_9817
Rhiz_Paulinella_chromatophora
Bact_Cyanobacteria_Synechococcus_sp_PCC_7335
Bact_Tene_Acholeplasma_laidlawii
Bact_Dfrb_Deferribacter_desulfuricans
-1/1
-/-/-
Bact_Fibrobacteres_Pyrinomonas_methylaliphatogenes
Bact_Arch_uncultured_crenarchaeote
-/1/0.98
-/-/-
Bact_Cf_Sphaerobacter_thermophilus
Bact_Nispir_Thermodesulfovibrio_yellowstonii
-/1/1
-/38/48
Bact_Chrysogen_Desulfurispirillum_indicum
Bact_Nispin_Nitrospina_sp_SCGC_AAA799_C22
0.60/0.99/0.99
35/49/50
Bact_Prot_Geobacter_metallireducens
Cil_Undescribed_sp
Bact_Tdsb_Thermodesulfatator_indicus
Bact_Elu_Endomicrobium_proavitum
Bact_Dictyoglomas_Dictyoglomus_thermophilum
Bact_Thermotogae_Kosmotoga_olearia
Bact_Cobi_Ignavibacterium_album
0.2

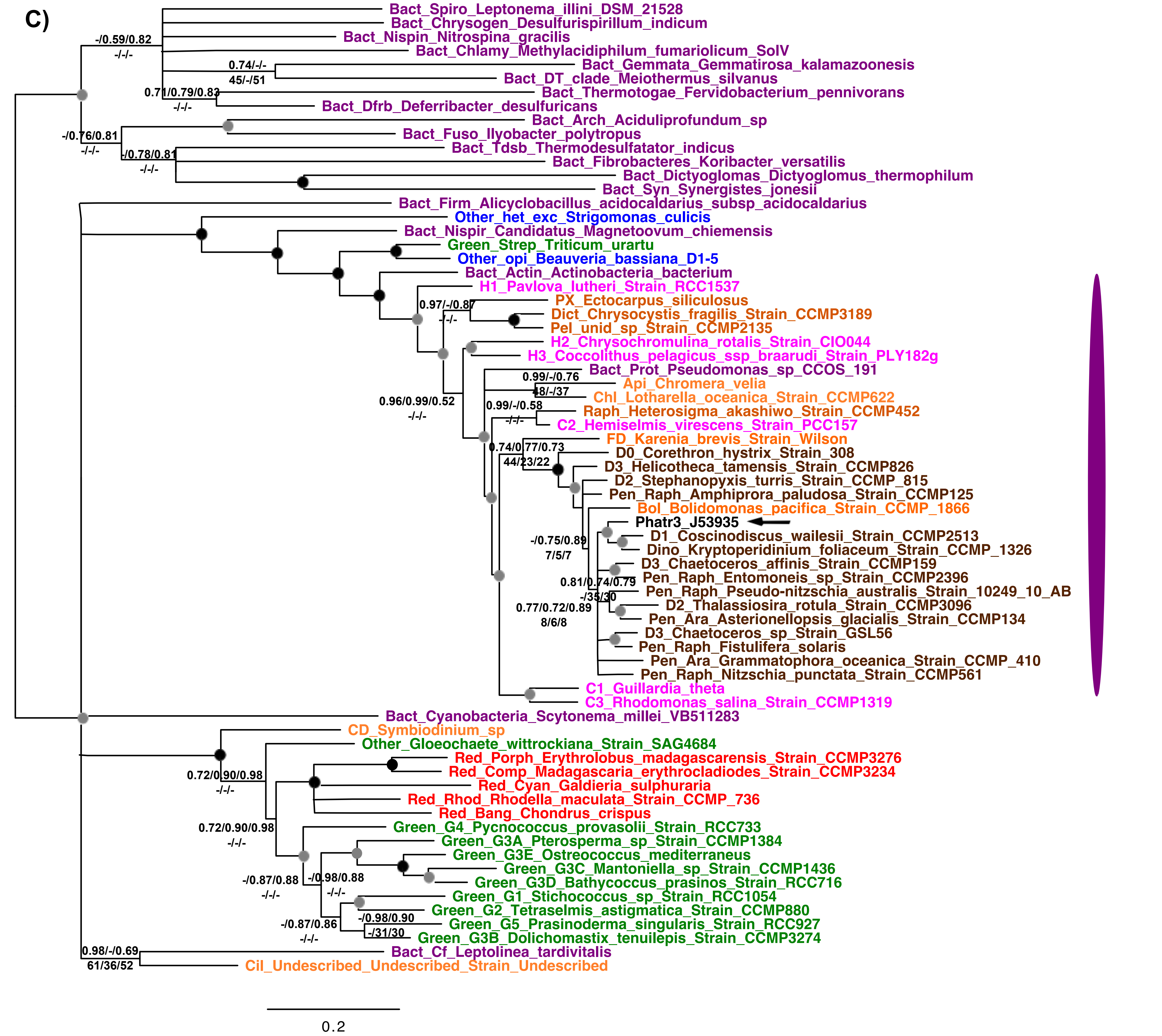
C)
Bact_Spiro_Leptonema_illini_DSM_21528
Bact_Chrysogen_Desulfurispirillum_indicum
Bact_Nispin_Nitrospina_gracilis
Bact_Chlamy_Methylacidiphilum_fumariolicum_SolV
Bact_Gemmata_Gemmatirosa_kalamazoonesis
Bact_DT_clade_Meiothermus_silvanus
Bact_Thermotogae_Fervidobacterium_pennivorans
Bact_Dfrb_Deferribacter_desulfuricans
Bact_Arch_Aciduliprofundum_sp
Bact_Fuso_Ilyobacter_polytropus
Bact_Tdsb_Thermodesulfatator_indicus
Bact_Fibrobacteres_Koribacter_versatilis
Bact_Dictyoglomas_Dictyoglomus_thermophilum
Bact_Syn_Synergistes_jonesii
Bact_Firm_Alicyclobacillus_acidocaldarius_subsp_acidocaldarius
Other_het_exc_Strigomonas_culicis
Bact_Nispir_Candidatus_Magnetoovum_chiemensis
Green_Strep_Triticum_urartu
Other_opi_Beauveria_bassiana_D1-5
Bact_Actin_Actinobacteria_bacterium
H1_Pavlova_lutheri_Strain_RCC1537
PX_Ectocarpus_siliculosus
Dict_Chrysocystis_fragilis_Strain_CCMP3189
Pel_unid_sp_Strain_CCMP2135
H2_Chrysochromulina_rotalis_Strain_CIO044
H3_Coccolithus_pelagicus_ssp_braarudi_Strain_PLY182g
Bact_Prot_Pseudomonas_sp_CCOS_191
Api_Chromera_velia
Chl_Lotharella_oceanica_Strain_CCMP622
Raph_Heterosigma_akashiwo_Strain_CCMP452
C2_Hemiselmis_virescens_Strain_PCC157
FD_Karenia_brevis_Strain_Wilson
D0_Corethron_hystrix_Strain_308
D3_Helicotheca_tamensis_Strain_CCMP826
D2_Stephanopyxis_turris_Strain_CCMP_815
Pen_Raph_Amphiprora_paludosa_Strain_CCMP125
Bol_Bolidomonas_pacifica_Strain_CCMP_1866
Phatr3_J53935
D1_Coscinodiscus_wailesii_Strain_CCMP2513
Dino_Kryptoperidinium_foliaceum_Strain_CCMP_1326
D3_Chaetoceros_affinis_Strain_CCMP159
Pen_Raph_Entomoneis_sp_Strain_CCMP2396
Pen_Raph_Pseudo-nitzschia_australis_Strain_10249_10_AB
D2_Thalassiosira_rotula_Strain_CCMP3096
Pen_Ara_Asterionellopsis_glacialis_Strain_CCMP134
D3_Chaetoceros_sp_Strain_GSL56
Pen_Raph_Fistulifera_solaris
Pen_Ara_Grammatophora_oceanica_Strain_CCMP_410
Pen_Raph_Nitzschia_punctata_Strain_CCMP561
C1_Guillardia_theta
C3_Rhodomonas_salina_Strain_CCMP1319
Bact_Cyanobacteria_Scytonema_millei_VB511283
CD_Symbiodinium_sp
Other_Gloeochaete_wittrockiana_Strain_SAG4684
Red_Porph_Erythrolobus_madagascarensis_Strain_CCMP3276
Red_Comp_Madagascaria_erythrocladiodes_Strain_CCMP3234
Red_Cyan_Galdieria_sulphuraria
Red_Rhod_Rhodella_maculata_Strain_CCMP_736
Red_Bang_Chondrus_crispus
Green_G4_Pycnococcus_provasolii_Strain_RCC733
Green_G3A_Pterosperma_sp_Strain_CCMP1384
Green_G3E_Ostreococcus_mediterraneus
Green_G3C_Mantoniella_sp_Strain_CCMP1436
Green_G3D_Bathycoccus_prasinos_Strain_RCC716
Green_G1_Stichococcus_sp_Strain_RCC1054
Green_G2_Tetraselmis_astigmatica_Strain_CCMP880
Green_G5_Prasinoderma_singularis_Strain_RCC927
Green_G3B_Dolichomastix_tenuilepis_Strain_CCMP3274
Bact_Cf_Leptolinea_tardivitalis
Cil_Undescribed_Undescribed_Strain_Undescribed
-/0.59/0.82
-/-/-
0.74/-/-
45/-/51
0.71/0.79/0.83
-/-/-
-/0.76/0.81
-/-/-
-/0.78/0.81
-/-/-
0.97/-/0.87
-/-/-
0.96/0.99/0.52
-/-/-
0.99/-/0.76
48/-/37
0.99/-/0.58
-/-/-
0.74/0.77/0.73
44/23/22
-/0.75/0.89
7/5/7
0.81/0.74/0.79
-/35/30
0.77/0.72/0.89
8/6/8
0.72/0.90/0.98
-/-/-
0.72/0.90/0.98
-/-/-
-/0.87/0.88
-/-/-
-/0.98/0.88
-/-/-
-/0.87/0.86
-/-/-
-/0.98/0.90
-/31/30
0.98/-/0.69
61/36/52
0.2

D)

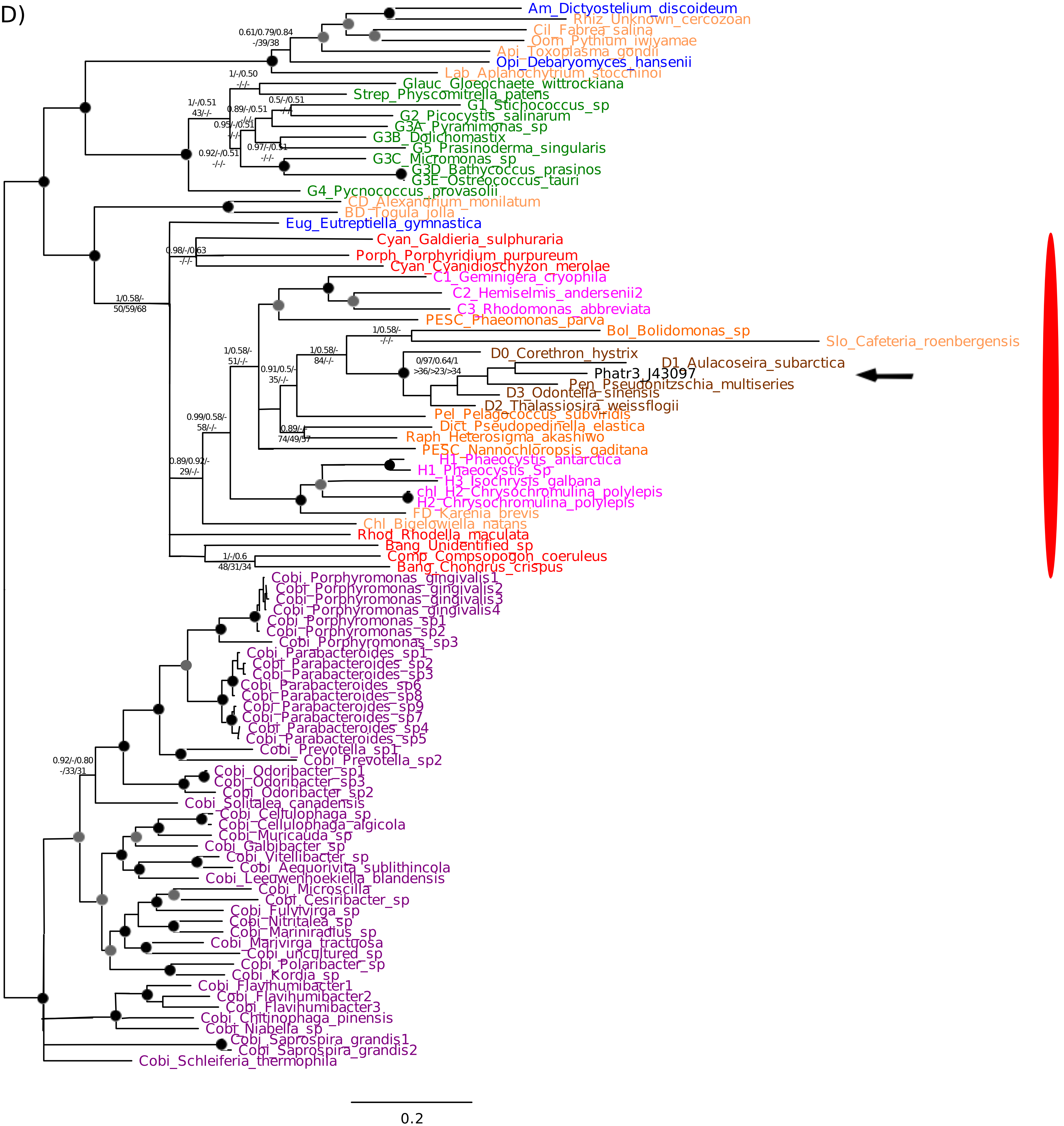
Am_Dictyostelium_discoideum
Rhiz_Unknown_cercozoan
Cil_Fabrea_salina
Oom_Pythium_iwiyamae
Api_Toxoplasma_gondii
Opi_Debaryomyces_hansenii
Lab_Aplanochytrium_stocchinoi
Glauc_Gloeochaete_wittrockiana
Strep_Physcomitrella_patens
G1_Stichococcus_sp
G2_Picocystis_salinarum
G3A_Pyramimonas_sp
G3B_Dolichomastix
G5_Prasinoderma_singularis
G3C_Micromonas_sp
G3D_Bathycoccus_prasinos
G3E_Ostreococcus_tauri
G4_Pycnococcus_provasolii
CD_Alexandrium_monilatum
BD_Togula_jolla
Eug_Eutreptiella_gymnastica
Cyan_Galdieria_sulphuraria
Porph_Porphyridium_purpureum
Cyan_Cyanidioschyzon_merolae
C1_Geminigera_cryophila
C2_Hemiselmis_andersenii2
C3_Rhodomonas_abbreviata
PESC_Phaeomonas_parva
Bol_Bolidomonas_sp
Slo_Cafeteria_roenbergensis
D0_Corethron_hystrix
D1_Aulacoseira_subarctica
Phatr3_J43097
Pen_Pseudonitzschia_multiseries
D3_Odontella_sinensis
D2_Thalassiosira_weissflogii
Pel_Pelagococcus_subviridis
Dict_Pseudopedinella_elastica
Raph_Heterosigma_akashiwo
PESC_Nannochloropsis_gaditana
H1_Phaeocystis_antarctica
H1_Phaeocystis_Sp
H3_Isochrysis_galbana
chl_H2_Chrysochromulina_polylepis
H2_Chrysochromulina_polylepis
FD_Karenia_brevis
Chl_Bigelowiella_natans
Rhod_Rhodella_maculata
Bang_Unidentified_sp
Comp_Compsopogon_coeruleus
Bang_Chondrus_crispus
Cobi_Porphyromonas_gingivalis1
Cobi_Porphyromonas_gingivalis2
Cobi_Porphyromonas_gingivalis3
Cobi_Porphyromonas_gingivalis4
Cobi_Porphyromonas_sp1
Cobi_Porphyromonas_sp2
Cobi_Porphyromonas_sp3
Cobi_Parabacteroides_sp1
Cobi_Parabacteroides_sp2
Cobi_Parabacteroides_sp3
Cobi_Parabacteroides_sp6
Cobi_Parabacteroides_sp8
Cobi_Parabacteroides_sp9
Cobi_Parabacteroides_sp7
Cobi_Parabacteroides_sp4
Cobi_Parabacteroides_sp5
Cobi_Prevotella_sp1
Cobi_Prevotella_sp2
Cobi_Odoribacter_sp1
Cobi_Odoribacter_sp3
Cobi_Odoribacter_sp2
Cobi_Solitalea_canadensis
Cobi_Cellulophaga_sp
Cobi_Cellulophaga_algicola
Cobi_Muricauda_sp
Cobi_Galbibacter_sp
Cobi_Vitellibacter_sp
Cobi_Aequorivita_sublithincola
Cobi_Leeuwenhoekiella_blandensis
Cobi_Microscilla
Cobi_Cesiribacter_sp
Cobi_Fulvivirga_sp
Cobi_Nitritalea_sp
Cobi_Mariniradius_sp
Cobi_Marivirga_tractuosa
Cobi_uncultured_sp
Cobi_Polaribacter_sp
Cobi_Kordia_sp
Cobi_Flavihumibacter1
Cobi_Flavihumibacter2
Cobi_Flavihumibacter3
Cobi_Chitinophaga_pinensis
Cobi_Niabella_sp
Cobi_Saprospira_grandis1
Cobi_Saprospira_grandis2
Cobi_Schleiferia_thermophila
0.61/0.79/0.84
-/39/38
1/-/0.50
-/-/-
1/-/0.51
43/-/-
0.5/-/0.51
0.89/-/0.51
0.95/-/0.51
0.92/-/0.51
0.97/-/0.51
0.98/-/0.63
1/0.58/-
50/59/68
1/0.58/-
1/0.58/-
84/-/-
0/97/0.64/1
>36/>23/>34
1/0.58/-
51/-/-
0.91/0.5/-
35/-/-
0.99/0.58/-
58/-/-
0.89/-/-
74/49/57
0.89/0.92/-
29/-/-
1/-/0.6
48/31/34
0.92/-/0.80
-/33/31
0.2

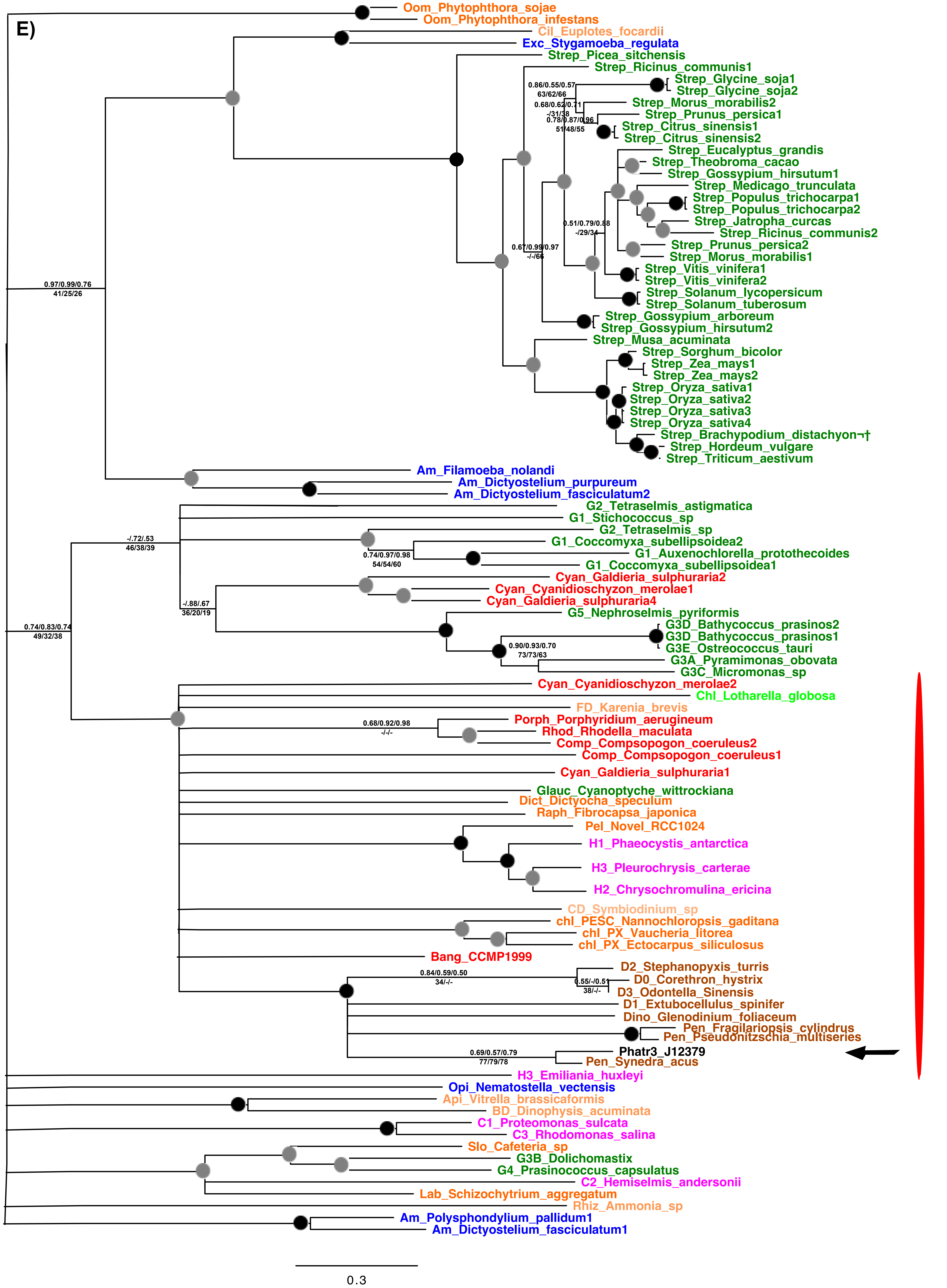
E)
Oom_Phytophthora_sojae
Oom_Phytophthora_infestans
Cil_Euplotes_focardii
Exc_Stygamoeba_regulata
Strep_Picea_sitchensis
Strep_Ricinus_communis1
0.86/0.55/0.57
63/62/66
Strep_Glycine_soja1
Strep_Glycine_soja2
0.68/0.62/0.71
-/31/38
Strep_Morus_morabilis2
0.78/0.87/0.96
51/48/55
Strep_Prunus_persica1
Strep_Citrus_sinensis1
Strep_Citrus_sinensis2
Strep_Eucalyptus_grandis
Strep_Theobroma_cacao
Strep_Gossypium_hirsutum1
Strep_Medicago_truncullata
Strep_Populus_trichocarpa1
Strep_Populus_trichocarpa2
Strep_Jatropha_curcas
0.51/0.79/0.88
-/29/34
Strep_Ricinus_communis2
0.67/0.99/0.97
-/-/66
Strep_Prunus_persica2
Strep_Morus_morabilis1
0.97/0.99/0.76
41/25/26
Strep_Vitis_vinifera1
Strep_Vitis_vinifera2
Strep_Solanum_lycopersicum
Strep_Solanum_tuberosum
Strep_Gossypium_arboreum
Strep_Gossypium_hirsutum2
Strep_Musa_acuminata
Strep_Sorghum_bicolor
Strep_Zea_mays1
Strep_Zea_mays2
Strep_Oryza_sativa1
Strep_Oryza_sativa2
Strep_Oryza_sativa3
Strep_Oryza_sativa4
Strep_Brachypodium_distachyon¬†
Strep_Hordeum_vulgare
Strep_Triticum_aestivum
Am_Filamoeba_nolandi
Am_Dictyostelium_purpureum
Am_Dictyostelium_fasciculatum2
G2_Tetraselmis_astigmatica
-/.72/.53
46/38/39
G1_Stichococcus_sp
G2_Tetraselmis_sp
G1_Coccomyxa_subellipsoidea2
0.74/0.97/0.98
54/54/60
G1_Auxenochlorella_protothecoides
G1_Coccomyxa_subellipsoidea1
Cyan_Galdieria_sulphuraria2
Cyan_Cyanidioschyzon_merolae1
-/.88/.67
36/20/19
Cyan_Galdieria_sulphuraria4
G5_Nephroselmis_pyriformis
0.74/0.83/0.74
49/32/38
G3D_Bathycoccus_prasinos2
G3D_Bathycoccus_prasinos1
0.90/0.93/0.70
73/73/63
G3E_Ostreococcus_tauri
G3A_Pyramimonas_obovata
G3C_Micromonas_sp
Cyan_Cyanidioschyzon_merolae2
Chl_Lotharella_globosa
FD_Karenia_brevis
0.68/0.92/0.98
-/-/-
Porph_Porphyridium_aerugineum
Rhod_Rhodella_maculata
Comp_Compsopogon_coeruleus2
Comp_Compsopogon_coeruleus1
Cyan_Galdieria_sulphuraria1
Glauc_Cyanoptyche_wittrockiana
Dict_Dictyocha_speculum
Raph_Fibrocapsa_japonica
Pel_Novel_RCC1024
H1_Phaeocystis_antarctica
H3_Pleurochrysis_carterae
H2_Chrysochromulina_ericina
CD_Symbiodinium_sp
chl_PESC_Nannochloropsis_gaditana
chl_PX_Vaucheria_litorea
chl_PX_Ectocarpus_siliculosus
Bang_CCMP1999
D2_Stephanopyxis_turris
0.84/0.59/0.50
34/-/-
0.55/-/0.51
38/-/-
D0_Corethron_hystrix
D3_Odontella_Sinensis
D1_Extubocellulus_spinifer
Dino_Glenodinium_foliaceum
Pen_Fragilariopsis_cylindrus
Pen_Pseudonitzschia_multiseries
0.69/0.57/0.79
77/79/78
Phatr3_J12379
Pen_Synedra_acus
H3_Emiliania_huxleyi
Opi_Nematostella_vectensis
Api_Vitrella_brassicaformis
BD_Dinophysis_acuminata
C1_Proteomonas_sulcata
C3_Rhodomonas_salina
Slo_Cafeteria_sp
G3B_Dolichomastix
G4_Prasinococcus_capsulatus
C2_Hemiselmis_andersonii
Lab_Schizochytrium_aggregatum
Rhiz_Ammonia_sp
Am_Polysphondylium_pallidum1
Am_Dictyostelium_fasciculatum1
0.3

F)

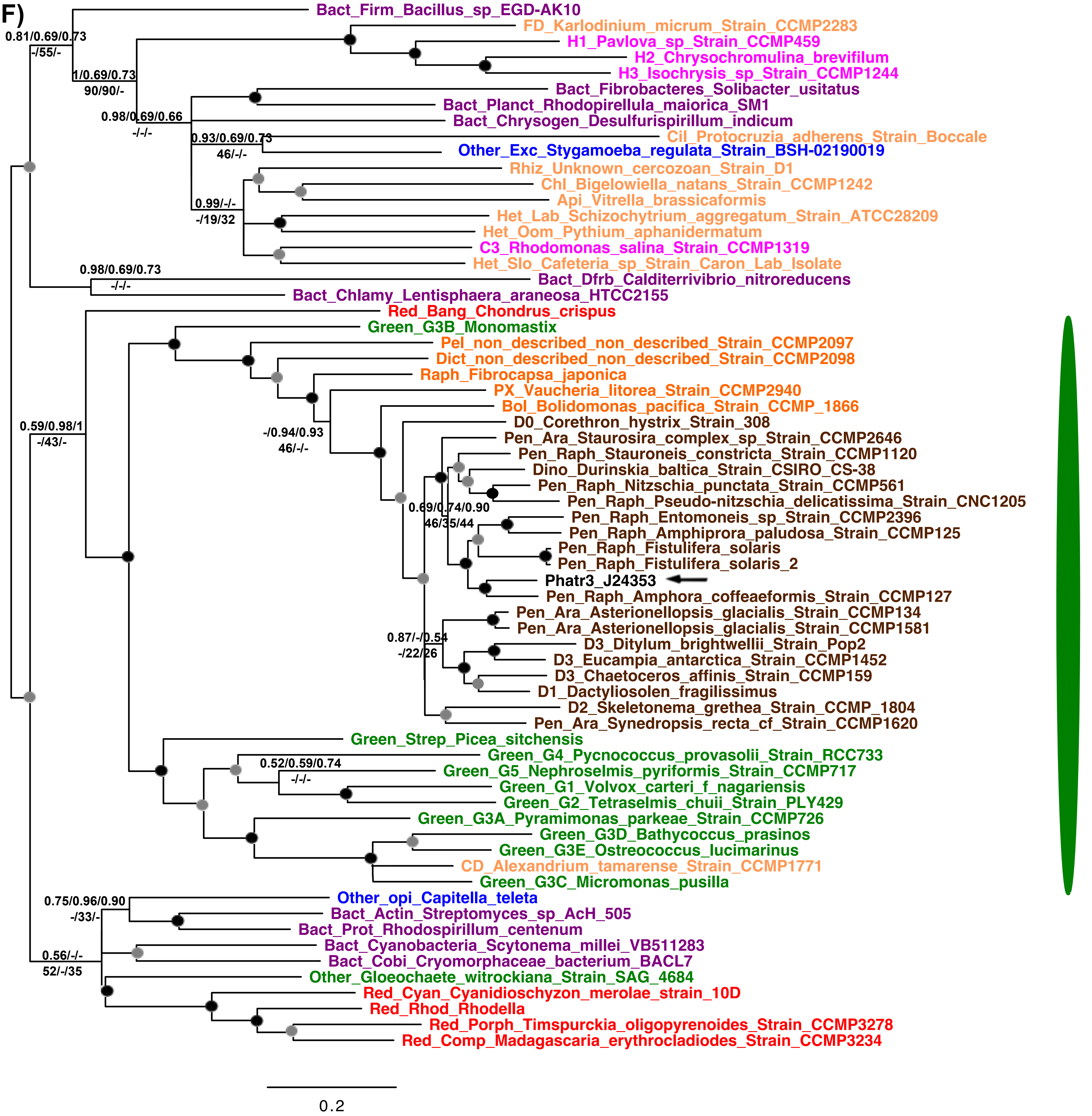

Bact_Firm_Bacillus_sp_EGD-AK10
FD_Karlodinium_micrum_Strain_CCMP2283
H1_Pavlova_sp_Strain_CCMP459
H2_Chrysochromulina_brevifilum
H3_Isochrysis_sp_Strain_CCMP1244
Bact_Fibrobacteres_Solibacter_usitatus
Bact_Planct_Rhodopirellula_maiorica_SM1
Bact_Chrysogen_Desulfurispirillum_indicum
Cil_Protocruzia_adherens_Strain_Boccale
Other_Exc_Stygamoeba_regulata_Strain_BSH-02190019
Rhiz_Unknown_cercozoan_Strain_D1
Chl_Bigelowiella_natans_Strain_CCMP1242
Api_Vitrella_brassicaformis
Het_Lab_Schizochytrium_aggregatum_Strain_ATCC28209
Het_Oom_Pythium_aphanidermatum
C3_Rhodomonas_salina_Strain_CCMP1319
Het_Slo_Cafeteria_sp_Strain_Caron_Lab_Isolate
Bact_Dfrb_Calditerrivibrio_nitroreducens
Bact_Chlamy_Lentisphaera_araneosa_HTCC2155
Red_Bang_Chondrus_crispus
Green_G3B_Monomastix
Pel_non_described_non_described_Strain_CCMP2097
Dict_non_described_non_described_Strain_CCMP2098
Raph_Fibrocapsa_japonica
PX_Vaucheria_litorea_Strain_CCMP2940
Bol_Bolidomonas_pacifica_Strain_CCMP_1866
D0_Corethron_hystrix_Strain_308
Pen_Ara_Staurosira_complex_sp_Strain_CCMP2646
Pen_Raph_Stauroneis_constricta_Strain_CCMP1120
Dino_Durinskia_baltica_Strain_CSIRO_CS-38
Pen_Raph_Nitzschia_punctata_Strain_CCMP561
Pen_Raph_Pseudo-nitzschia_delicatissima_Strain_CNC1205
Pen_Raph_Entomoneis_sp_Strain_CCMP2396
Pen_Raph_Amphiprora_paludosa_Strain_CCMP125
Pen_Raph_Fistulifera_solaris
Pen_Raph_Fistulifera_solaris_2
Phatr3_J24353
Pen_Raph_Amphora_coffeaeformis_Strain_CCMP127
Pen_Ara_Asterionellopsis_glacialis_Strain_CCMP134
Pen_Ara_Asterionellopsis_glacialis_Strain_CCMP1581
D3_Ditylum_brightwellii_Strain_Pop2
D3_Eucampia_antarctica_Strain_CCMP1452
D3_Chaetoceros_affinis_Strain_CCMP159
D1_Dactyliosolen_fragilissimus
D2_Skeletonema_grethea_Strain_CCMP_1804
Pen_Ara_Synedropsis_recta_cf_Strain_CCMP1620
Green_Strep_Picea_sitchensis
Green_G4_Pycnococcus_provasolii_Strain_RCC733
Green_G5_Nephroselmis_pyriformis_Strain_CCMP717
Green_G1_Volvox_carteri_f_nagariensis
Green_G2_Tetraselmis_chuii_Strain_PLY429
Green_G3A_Pyramimonas_parkeae_Strain_CCMP726
Green_G3D_Bathycoccus_prasinos
Green_G3E_Ostreococcus_lucimarinus
CD_Alexandrium_tamarense_Strain_CCMP1771
Green_G3C_Micromonas_pusilla
Other_opi_Capitella_teleta
Bact_Actin_Streptomyces_sp_AcH_505
Bact_Prot_Rhodospirillum_centenum
Bact_Cyanobacteria_Scytonema_millei_VB511283
Bact_Cobi_Cryomorphaceae_bacterium_BACL7
Other_Gloeochaete_witrockiana_Strain_SAG_4684
Red_Cyan_Cyanidioschyzon_merolae_strain_10D
Red_Rhod_Rhodella
Red_Porph_Timspurckia_oligopyrenoides_Strain_CCMP3278
Red_Comp_Madagascaria_erythrocladiodes_Strain_CCMP3234
0.81/0.69/0.73
-/55/-
1/0.69/0.73
90/90/-
0.98/0.69/0.66
-/-/-
0.93/0.69/0.73
46/-/-
0.99/-/-
-/19/32
0.98/0.69/0.73
-/-/-
0.59/0.98/1
-/43/-
-/0.94/0.93
46/-/-
0.69/0.74/0.90
46/35/44
0.87/-/0.54
-/22/26
0.52/0.59/0.74
-/-/-
0.75/0.96/0.90
-/33/-
0.56/-/-
52/-/35
0.2

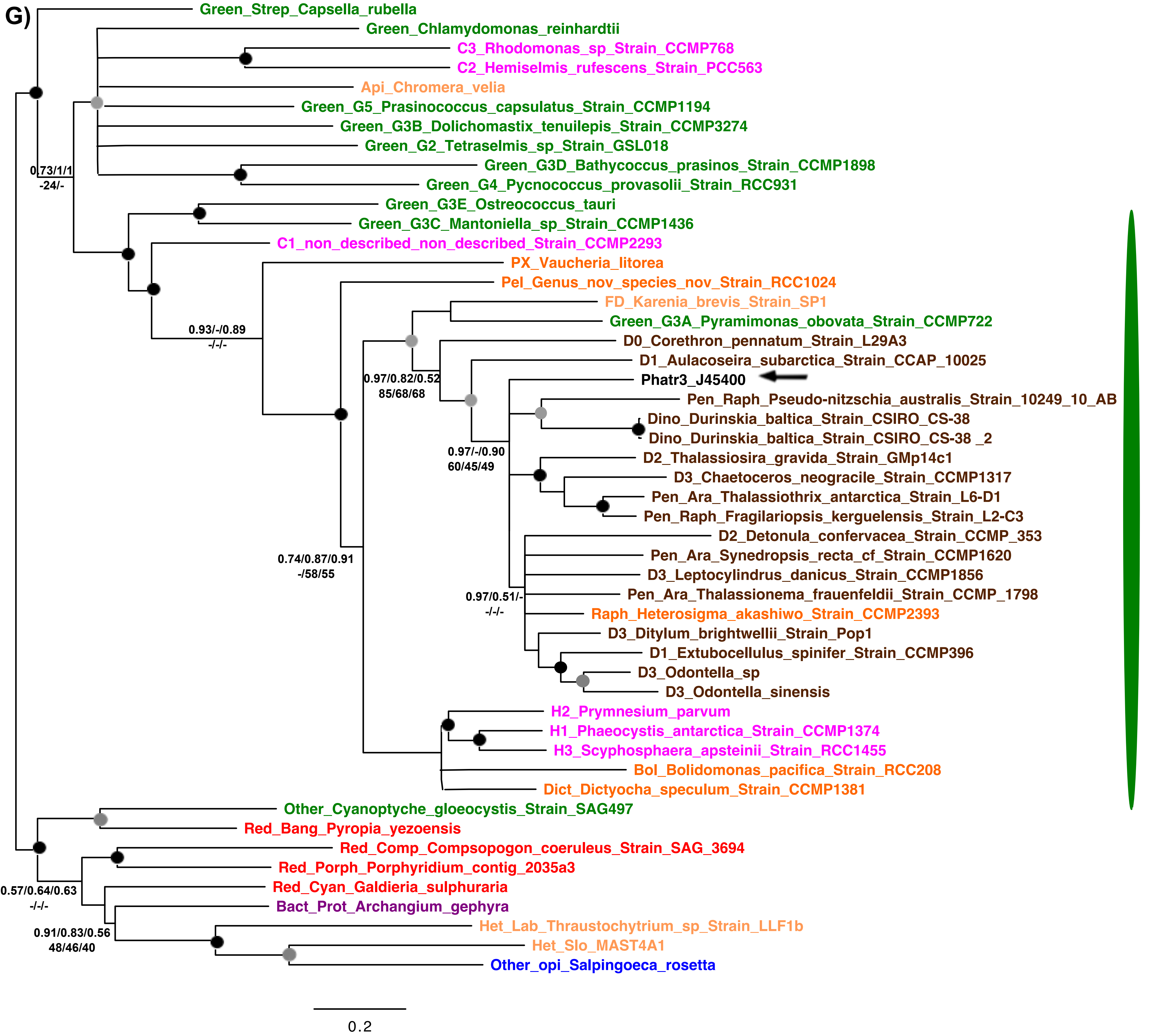

G)
Green_Strep_Capsella_rubella
Green_Chlamydomonas_reinhardtii
C3_Rhodomonas_sp_Strain_CCMP768
C2_Hemiselmis_rufescens_Strain_PCC563
Api_Chromera_velia
Green_G5_Prasinococcus_capsulatus_Strain_CCMP1194
Green_G3B_Dolichomastix_tenuilepis_Strain_CCMP3274
Green_G2_Tetraselmis_sp_Strain_GSL018
Green_G3D_Bathycoccus_prasinos_Strain_CCMP1898
Green_G4_Pycnococcus_provasolii_Strain_RCC931
0.73/1/1
-24/-
Green_G3E_Ostreococcus_tauri
Green_G3C_Mantoniella_sp_Strain_CCMP1436
C1_non_described_non_described_Strain_CCMP2293
PX_Vaucheria_litorea
Pel_Genus_nov_species_nov_Strain_RCC1024
FD_Karenia_brevis_Strain_SP1
Green_G3A_Pyramimonas_obovata_Strain_CCMP722
0.93/-/0.89
-/-/-
D0_Corethron_pennatum_Strain_L29A3
D1_Aulacoseira_subarctica_Strain_CCAP_10025
0.97/0.82/0.52
85/68/68
Phatr3_J45400
Pen_Raph_Pseudo-nitzschia_australis_Strain_10249_10_AB
Dino_Durinskia_baltica_Strain_CSIRO_CS-38
Dino_Durinskia_baltica_Strain_CSIRO_CS-38 _2
0.97/-/0.90
60/45/49
D2_Thalassiosira_gravida_Strain_GMp14c1
D3_Chaetoceros_neogracile_Strain_CCMP1317
Pen_Ara_Thalassiothrix_antarctica_Strain_L6-D1
Pen_Raph_Fragilariopsis_kerguelensis_Strain_L2-C3
D2_Detonula_confervacea_Strain_CCMP_353
0.74/0.87/0.91
-/58/55
Pen_Ara_Synedropsis_recta_cf_Strain_CCMP1620
D3_Leptocylindrus_danicus_Strain_CCMP1856
0.97/0.51/-
-/-/-
Pen_Ara_Thalassionema_frauenfeldii_Strain_CCMP_1798
Raph_Heterosigma_akashiwo_Strain_CCMP2393
D3_Ditylum_brightwellii_Strain_Pop1
D1_Extubocellulus_spinifer_Strain_CCMP396
D3_Odontella_sp
D3_Odontella_sinensis
H2_Prymnesium_parvum
H1_Phaeocystis_antarctica_Strain_CCMP1374
H3_Scyphosphaera_apsteinii_Strain_RCC1455
Bol_Bolidomonas_pacifica_Strain_RCC208
Dict_Dictyocha_speculum_Strain_CCMP1381
Other_Cyanoptyche_gloeocystis_Strain_SAG497
Red_Bang_Pyropia_yezoensis
Red_Comp_Compsopogon_coeruleus_Strain_SAG_3694
Red_Porph_Porphyridium_contig_2035a3
0.57/0.64/0.63
-/-/-
Red_Cyan_Galdieria_sulphuraria
Bact_Prot_Archangium_gephyra
0.91/0.83/0.56
48/46/40
Het_Lab_Thraustochytrium_sp_Strain_LLF1b
Het_Slo_MAST4A1
Other_opi_Salpingoeca_rosetta
0.2

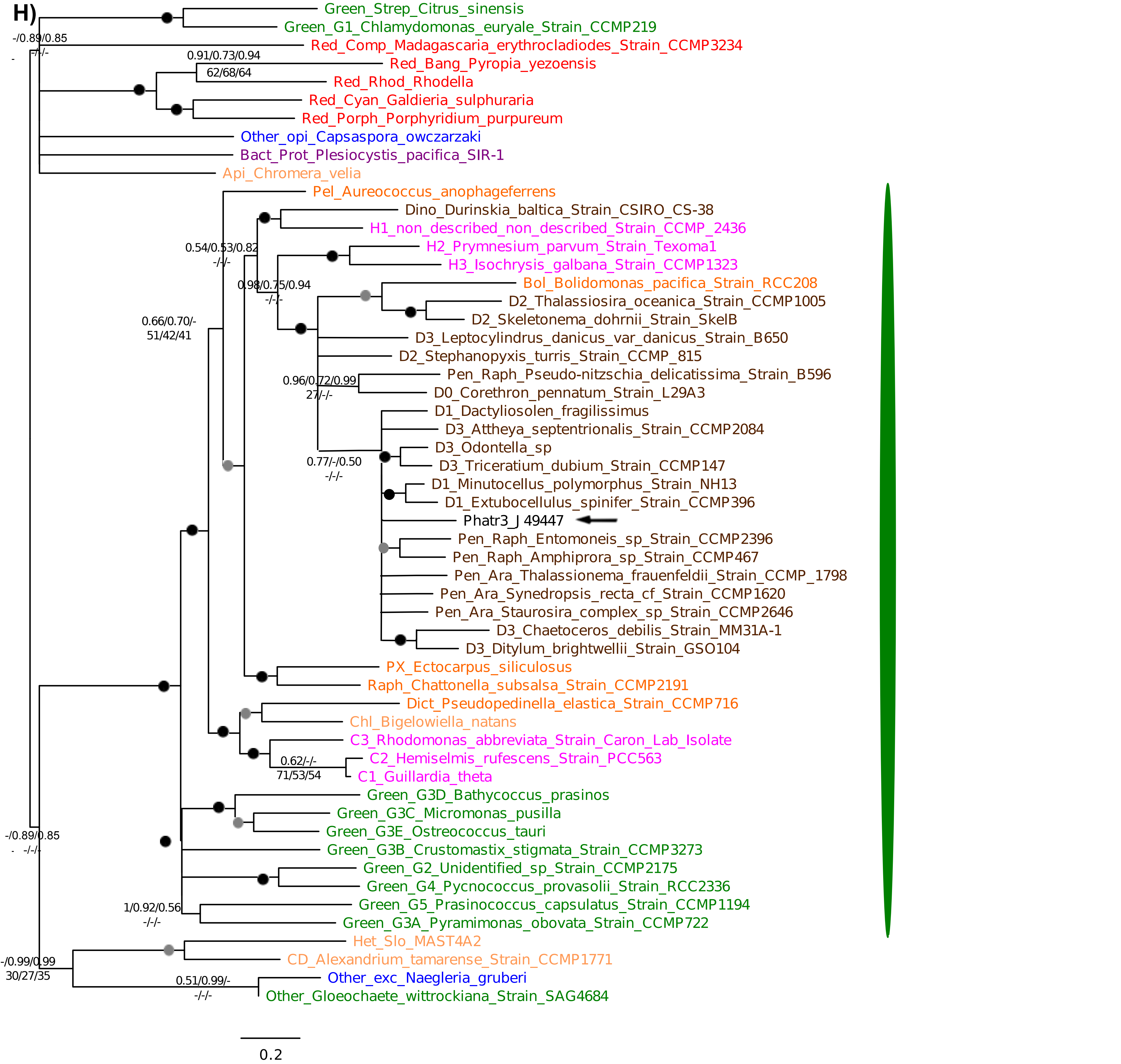
H)
Green_Strep_Citrus_sinensis
Green_G1_Chlamydomonas_euryale_Strain_CCMP219
Red_Comp_Madagascaria_erythrocladiodes_Strain_CCMP3234
Red_Bang_Pyropia_yezoensis
Red_Rhod_Rhodella
Red_Cyan_Galdieria_sulphuraria
Red_Porph_Porphyridium_purpureum
Other_opi_Capsaspora_owczarzaki
Bact_Prot_Plesiocystis_pacifica_SIR-1
Api_Chromera_velia
Pel_Aureococcus_anophageferrens
Dino_Durinskia_baltica_Strain_CSIRO_CS-38
H1_non_described_non_described_Strain_CCMP_2436
H2_Prymnesium_parvum_Strain_Texoma1
H3_Isochrysis_galbana_Strain_CCMP1323
Bol_Bolidomonas_pacifica_Strain_RCC208
D2_Thalassiosira_oceanica_Strain_CCMP1005
D2_Skeletonema_dohrnii_Strain_SkelB
D3_Leptocylindrus_danicus_var_danicus_Strain_B650
D2_Stephanopyxis_turris_Strain_CCMP_815
Pen_Raph_Pseudo-nitzschia_delicatissima_Strain_B596
D0_Corethron_pennatum_Strain_L29A3
D1_Dactyliosolen_fragilissimus
D3_Attheya_septentrionalis_Strain_CCMP2084
D3_Odontella_sp
D3_Triceratium_dubium_Strain_CCMP147
D1_Minutocellus_polymorphus_Strain_NH13
D1_Extubocellulus_spinifer_Strain_CCMP396
Phatr3_J 49447
Pen_Raph_Entomoneis_sp_Strain_CCMP2396
Pen_Raph_Amphiprora_sp_Strain_CCMP467
Pen_Ara_Thalassionema_frauenfeldii_Strain_CCMP_1798
Pen_Ara_Synedropsis_recta_cf_Strain_CCMP1620
Pen_Ara_Staurosira_complex_sp_Strain_CCMP2646
D3_Chaetoceros_debilis_Strain_MM31A-1
D3_Ditylum_brightwellii_Strain_GSO104
PX_Ectocarpus_siliculosus
Raph_Chattonella_subsalsa_Strain_CCMP2191
Dict_Pseudopedinella_elastica_Strain_CCMP716
Chl_Bigelowiella_natans
C3_Rhodomonas_abbreviata_Strain_Caron_Lab_Isolate
C2_Hemiselmis_rufescens_Strain_PCC563
C1_Guillardia_theta
Green_G3D_Bathycoccus_prasinos
Green_G3C_Micromonas_pusilla
Green_G3E_Ostreococcus_tauri
Green_G3B_Crustomastix_stigmata_Strain_CCMP3273
Green_G2_Unidentified_sp_Strain_CCMP2175
Green_G4_Pycnococcus_provasolii_Strain_RCC2336
Green_G5_Prasinococcus_capsulatus_Strain_CCMP1194
Green_G3A_Pyramimonas_obovata_Strain_CCMP722
Het_Slo_MAST4A2
CD_Alexandrium_tamarense_Strain_CCMP1771
Other_exc_Naegleria_gruberi
Other_Gloeochaete_wittrockiana_Strain_SAG4684
-/0.89/0.85
- -/-/-
0.91/0.73/0.94
62/68/64
0.54/0.53/0.82
-/-/-
0.98/0.75/0.94
-/-/-
0.66/0.70/-
51/42/41
0.96/0.72/0.99
27/-/-
0.77/-/0.50
-/-/-
0.62/-/-
71/53/54
-/0.89/0.85
- -/-/-
1/0.92/0.56
-/-/-
-/0.99/0.99
30/27/35
0.51/0.99/-
-/-/-
0.2

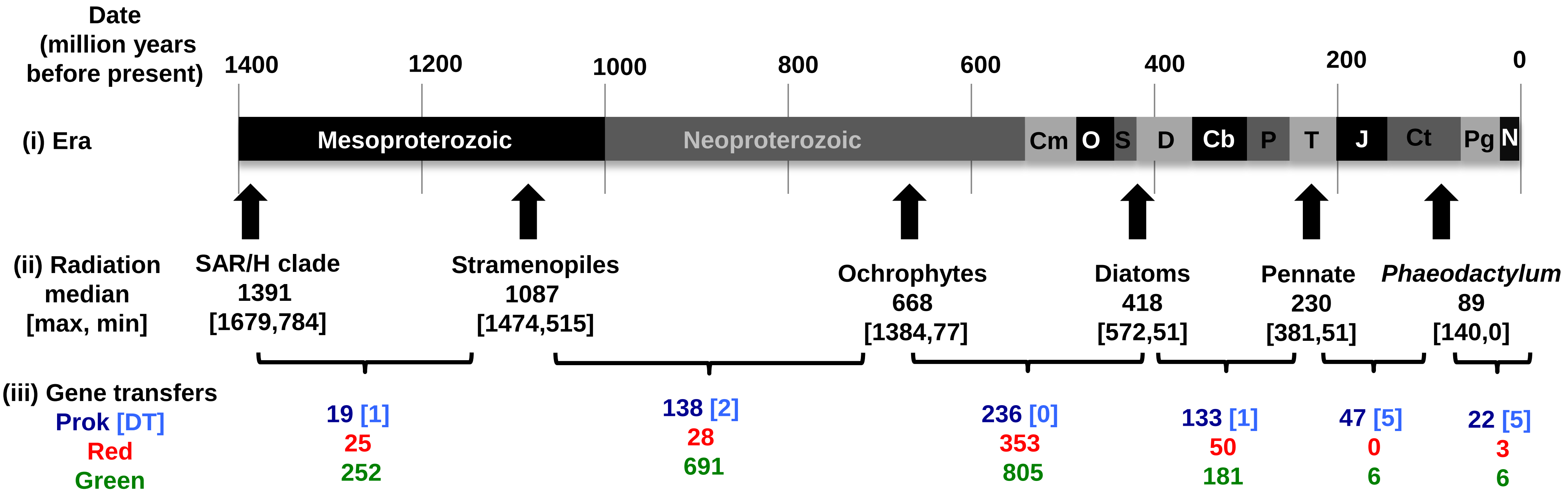
Date
(million years
before present)
1400
1200
1000
800
600
400
200
0
(i) Era
Mesoproterozoic
Neoproterozoic
Cm
O
S
D
Cb
P
T
J
Ct
Pg
N
(ii) Radiation
median
[max, min]
SAR/H clade
1391
[1679,784]
Stramenopiles
1087
[1474,515]
Ochrophytes
668
[1384,77]
Diatoms
418
[572,51]
Pennate
230
[381,51]
Phaeodactylum
89
[140,0]
(iii) Gene transfers
Prok [DT]
Red
Green
19 [1]
25
252
138 [2]
28
691
236 [0]
353
805
133 [1]
50
181
47 [5]
0
6
22 [5]
3
6
