# Supplementary material for: Integrative analysis of large scale transcriptome data draws a comprehensive landscape of Phaeodactylum tricornutum genome and evolutionary origin of diatoms
Source: Sci Rep. 2018 Mar 19;8:4834. doi: 10.1038/s41598-018-23106-x (PMC5859163; doi:10.1038/s41598-018-23106-x)
Supplement: Supplementary file 2 — File S2 [file 41598_2018_23106_MOESM2_ESM.pdf]

Integrative analysis of large scale transcriptome data draws a comprehensive landscape of *Phaeodactylum tricornutum* genome and evolutionary origin of diatoms

Achal Rastogi[1], Uma Maheswari[2], Richard G. Dorrell[1], Fabio Rocha Jimenez Vieira[1], Florian Maumus[3], Adam Kustka[4], James McCarthy[5], Andy E. Allen[5, 6], Paul Kersey[2], Chris Bowler[1*] and Leila Tirichine[1*]

Extended methods and description of data

Data generation and mining

Phaeodactylum tricornutum genome re-annotation (named as Phatr3) was done on the Phatr2 genome assembly (ASM15095v2)[1] using species-specific high throughput sequencing datasets as follows:

RNA-Seq

Multiple RNAseq libraries (91 libraries in total) were generated under different growth conditions and are being used for many functional studies[2]. The growth conditions used can be broadly divided into two major categories: 1) Nitrogen availability, which include 30 RNAseq libraries generated using Illumina platform (Bio-project accession no. PRJNA311568; Bio-sample accession numbers SAMN04488978-SAMN04489007), and 2) Iron availability, includes 49 libraries of RNA-Seq generated using SoLiD sequencing technology (SRA: SRP069841) [2]. 12 more RNAseq libraries were generated including the wild type and alternative oxidase (Phatr2_bd1075) mutants [3]; Bio-sample accessions: SAMN06350641-SAMN06350652. More information about the culture conditions can be referred from File S2.

Culture and growth conditions (RNAseq libraries)

1) Nitrogen availability: Duplicate 2 L of *P. tricornutum* cultures, CCAP-1055, were grown in artificial seawater medium with f/2 nutrients, trace metals and vitamins. In place of nitrate, 880μM NH_4^+ was added as the sole nitrogen source. Cultures were stirred and bubbled with air, in diel light (14:10, L:D, 150μE m^{-2} s^{-1}) at 18°C. At mid-exponential phase growth (Fv/Fm ≥ 0.6, ~3 X 10^6 cells ml^{-1}), cells were collected by centrifugation (10 min, 700xg, 18°C), washed 3 times in nitrogen-free (N-free) media, then re-suspended in N-free media in duplicate 2L. After 2 hours, cultures were spiked with NO_3^- to a final concentration of 150μM. After 90 minutes in nitrate media, cells were collected by centrifugation, washed three times (see above) and pellets re-suspended in 20 ml N-free media, combined and then aliquoted at ~7.5 X 10^5 cells ml^{-1} into duplicate, 800 ml of standard media containing different nitrogen sources: *a.* no nitrogen, *b.* 300μM NH_4^+, *c.* 300μM NO_3^-, *d.* 300 μM NO_2^-, in 1L Roux culture bottles (Corning). All cultures were

incubated in constant light at 18°C with bubbled air for the duration of the time course. Culture flasks were racked on an angle enabling the bubbled air to thoroughly circulate the cells. Samples for RNAseq were collected in duplicate at 6 time points: *1)* mid-exponential phase growth on ammonium, *2)* 2 hours in N-free media, *3)* 90 minutes after NO_3^- addition, *4)* 15 minutes, *5)* 45 minutes and *6)* 18 hours (referred as Tend) after being aliquoted into the 4 N-conditions. The data has also been used in a following study [4].

2) Iron availability: The conditions are well described in [2].

3) Others: The conditions are well described in [3].

Expressed sequence tags (ESTs)

Along with multiple RNAseq libraries existing 13,828 non-redundant *P. tricornutum* ESTs [5,6] done in different growth conditions were also utilized. Other EST data used includes 93,206 diatom ESTs from dbEST [7].

Construction of a multi-sequence reference dataset

The description of the taxonomic divisions (The divisions are precisely stated in Table S4 as well).

- **Diatoms**, consisting of two separate pennate sub-categories (raphid and araphid species), and four centric categories. Dinotoms (i.e. dinoflagellates harbouring diatom endosymbionts) were included in this category, as previous studies have indicated that the diatom endosymbiont retains a large gene complement of its own [8].
- **Non-diatom stramenopiles**, consisting of six plastid-bearing (ochrophyte) and three aplastidic sub-categories. This taxonomic assembly, while it is paraphyletic with regard to diatoms, was chosen as it enables the distinction of genes (by comparison to the diatom sequences) that are uniquely shared between *Phaeodactylum* and other diatoms from genes that are more broadly evolutionarily conserved across the stramenopiles.
- **Non-stramenopile SAR clade** members, consisting of six alveolate and two rhizarian sub-categories. Plastid-bearing chlorarachniophytes were separated from other (aplastidic) rhizarians due to the extensive plastid-associated gene transfer that has occurred in this group [9,10]. Fucoxanthin-containing dinoflagellates were similarly separated from other dinoflagellate groups due to the additional gene transfers that have occurred in this group following the replacement of the original peridinin-containing plastid [11]. This taxonomic assembly was chosen as it enables the distinction of genes (by comparison to

the stramenopile sequences) that are uniquely shared between *Phaeodactylum* and other stramenopiles from genes that are more broadly evolutionarily conserved across the SAR clade.

- **CCTH members**, consisting of three cryptomonad and three haptophyte sub-categories. This group was used, despite ongoing controversy over its taxonomic significance [12], due to the evidence for extensive gene sharing between different members [13].
- **Green eukaryotic groups**, consisting of streptophytes, chlorophytes and glaucophytes. Glaucophytes were included in this group, as recent phylogenomic studies place them as more closely related to the green group than red algae [12,14]. The chlorophytes were divided into a much greater number of sub-categories (nine) than streptophytes (treated as a single sub-category) to investigate the assertion from previous studies for a gene transfer event between prasinophyte algae and diatoms [15].
- **Red algae**, divided into five sub-categories.
- **Amorphea**, divided into two excavate sub-categories and two unikont sub-categories. This group was used, despite ongoing uncertainty over whether it is monophyletic or paraphyletic to all other eukaryotes [16,17] due to the limited gene transfers previously proposed to have occurred between these groups and *P. tricornutum* [1]. As per the situation for chlorarachniophytes and rhizaria, plastid-bearing euglenids were separated from all other excavates, due to the extensive plastid-associated gene transfer associated with this group [18].
- **Prokaryotes**, divided into twenty-six categories. Bacteria and archaea were combined due to evidence for extensive gene transfer between these two groups [19,20], despite the distant evolutionary relationships between their nucleoid genomes.
- **Viruses**, as a single sub-category.

Assessment of the conservation and complex evolutionary origin of *P. tricornutum* genome

In this analysis we designed a compound protocol, based on primary and secondary BLAST top hit identity, using libraries modified to remove different combinations of sequences to trace the evolutionary history of different lineages. This pipeline allows the preliminary assessment of the magnitude, relative composition, and timing different types of gene transfers into the ancestors of *Phaeodactylum*. Specific gene transfer events within this dataset may require case-by-case verification using single-gene trees; however, we note a broad concordance in the evolutionary histories predicted by this analysis, and a previously published phylogenetic study of 770 plastid-targeted proteins conserved across the diatom lineage [21] (Fig. S9; File S3, Table S11).

Validation of “modified gene models” using RT-PCR

Total cellular RNA was extracted from approximately 30 ml late-log phase *P. tricornutum*, grown as described above, by phase extraction with Trizol (Thermo, France), followed by treatment using RNAse-free DNAse (Qiagen, France) and cleanup using an RNeasy column (Qiagen) as previously described [21]. RNA was verified to be free of residual DNA contamination by PCR using previously generated universal 18S rDNA primers [22]. cDNA was synthesized from 100 ng RNA-free DNA using a Maxima First cDNA synthesis kit (Thermo), and PCR was performed using the cDNA template and primers designed against the 5' and 3' ends of genes of interest using DreamTaq DNA polymerase (Thermo), per the manufacturers' instructions. Products were separated by electrophoresis on a 1%-agarose TAE gel containing 0.2 µg/ml ethidium bromide at 100V for 30 minutes, and visualized with a UV transilluminator. Representative products from each reaction were purified using PCR cleanup spin columns (Macherey-Nagel, France), and confirmed by Sanger sequencing (GATC, France) using both the forward and reverse PCR primers.

Homology detection

Functional annotation was performed by CLADE 2.7 and DAMA 3.1 algorithms, where CLADE scanned the entire Phatr3 genome (all the six possible frame translations without any type of trim) looking for conserved regions. Next, DAMA filtered and selected the 8235 most statistically significant protein architectures (domain arrangements). For the other 3998 genes, 3857 where annotated by CLADE best model approach. It is worth note that the best model predictions are less statistically significant then DAMA predictions [23-25].

References

1 Bowler, C. *et al.* The Phaeodactylum genome reveals the evolutionary history of diatom genomes. *Nature* **456**, 239-244, doi:nature07410 [pii]
10.1038/nature07410 (2008).

2 Smith, S. R. *et al.* Transcriptional Orchestration of the Global Cellular Response of a Model Pennate Diatom to Diel Light Cycling under Iron Limitation. *PLoS Genet* **12**, e1006490, doi:10.1371/journal.pgen.1006490 (2016).

3 Bailleul, B. *et al.* Energetic coupling between plastids and mitochondria drives CO2 assimilation in diatoms. *Nature* **524**, 366-369, doi:10.1038/nature14599 (2015).

4 Levering, J., Dupont, C. L., Allen, A. E., Palsson, B. O. & Zengler, K. Integrated Regulatory and Metabolic Networks of the Marine Diatom Phaeodactylum tricornutum Predict the Response to Rising CO2 Levels. *mSystems* **2**, doi:10.1128/mSystems.00142-16 (2017).

5 Maheswari, U. *et al.* Digital expression profiling of novel diatom transcripts provides insight into their biological functions. *Genome biology* **11**, R85, doi:gb-2010-11-8-r85 [pii]
10.1186/gb-2010-11-8-r85 (2010).

6 Maheswari, U., Mock, T., Armbrust, E. V. & Bowler, C. Update of the Diatom EST Database: a new tool for digital transcriptomics. *Nucleic acids research* **37**, D1001-1005, doi:gkn905 [pii]
10.1093/nar/gkn905 (2009).
7 Boguski, M. S., Lowe, T. M. & Tolstoshev, C. M. dbEST--database for "expressed sequence tags". *Nature genetics* **4**, 332-333, doi:10.1038/ng0893-332 (1993).
8 Hehenberger, E., Burki, F., Kolisko, M. & Keeling, P. J. Functional Relationship between a Dinoflagellate Host and Its Diatom Endosymbiont. *Mol Biol Evol* **33**, 2376-2390, doi:10.1093/molbev/msw109 (2016).
9 Archibald, J. M., Rogers, M. B., Toop, M., Ishida, K. & Keeling, P. J. Lateral gene transfer and the evolution of plastid-targeted proteins in the secondary plastid-containing alga Bigelowiella natans. *Proceedings of the National Academy of Sciences of the United States of America* **100**, 7678-7683, doi:10.1073/pnas.1230951100 (2003).
10 Curtis, B. A. *et al.* Algal genomes reveal evolutionary mosaicism and the fate of nucleomorphs. *Nature* **492**, 59-65, doi:10.1038/nature11681 (2012).
11 Burki, F. *et al.* Endosymbiotic gene transfer in tertiary plastid-containing dinoflagellates. *Eukaryot Cell* **13**, 246-255, doi:10.1128/EC.00299-13 (2014).
12 Burki, F. Mitochondrial Evolution: Going, Going, Gone. *Current biology : CB* **26**, R410-412, doi:10.1016/j.cub.2016.04.032 (2016).
13 Stiller, J. W. Toward an empirical framework for interpreting plastid evolution. *J Phycol* **50**, 462-471, doi:10.1111/jpy.12178 (2014).
14 Burki, F. *et al.* Re-evaluating the green versus red signal in eukaryotes with secondary plastid of red algal origin. *Genome biology and evolution* **4**, 626-635, doi:10.1093/gbe/evs049 (2012).
15 Moustafa, A. *et al.* Genomic footprints of a cryptic plastid endosymbiosis in diatoms. *Science* **324**, 1724-1726, doi:324/5935/1724 [pii]
10.1126/science.1172983 (2009).
16 Derelle, R. *et al.* Bacterial proteins pinpoint a single eukaryotic root. *Proceedings of the National Academy of Sciences of the United States of America* **112**, E693-699, doi:10.1073/pnas.1420657112 (2015).
17 He, D. *et al.* An alternative root for the eukaryote tree of life. *Current biology : CB* **24**, 465-470, doi:10.1016/j.cub.2014.01.036 (2014).
18 Maruyama, S., Suzaki, T., Weber, A. P., Archibald, J. M. & Nozaki, H. Eukaryote-to-eukaryote gene transfer gives rise to genome mosaicism in euglenids. *BMC evolutionary biology* **11**, 105, doi:10.1186/1471-2148-11-105 (2011).
19 Nelson, K. E. *et al.* Evidence for lateral gene transfer between Archaea and bacteria from genome sequence of Thermotoga maritima. *Nature* **399**, 323-329, doi:10.1038/20601 (1999).
20 Garcia-Vallve, S., Romeu, A. & Palau, J. Horizontal gene transfer in bacterial and archaeal complete genomes. *Genome Res* **10**, 1719-1725 (2000).
21 Dorrell, R. G. *et al.* Chimeric origins of ochrophytes and haptophytes revealed through an ancient plastid proteome. *Elife* **6**, doi:10.7554/eLife.23717 (2017).
22 Gachon, C. M. M. *et al.* The CCAP KnowledgeBase: linking protistan and cyanobacterial biological resources with taxonomic and molecular data. *Systematics and Biodiversity* **11**, 407-413, doi:10.1080/14772000.2013.859641 (2013).

23 Bernardes, J., Zaverucha, G., Vaquero, C. & Carbone, A. Improvement in Protein Domain Identification Is Reached by Breaking Consensus, with the Agreement of Many Profiles and Domain Co-occurrence. *PLoS Comput Biol* **12**, e1005038, doi:10.1371/journal.pcbi.1005038 (2016).

24 Bernardes, J. S., Vieira, F. R., Costa, L. M. & Zaverucha, G. Evaluation and improvements of clustering algorithms for detecting remote homologous protein families. *BMC bioinformatics* **16**, 34, doi:10.1186/s12859-014-0445-4 (2015).

25 Bernardes, J. S., Vieira, F. R., Zaverucha, G. & Carbone, A. A multi-objective optimization approach accurately resolves protein domain architectures. *Bioinformatics* **32**, 345-353, doi:10.1093/bioinformatics/btv582 (2016).
