# Supplementary material for: Integrative analysis of large scale transcriptome data draws a comprehensive landscape of Phaeodactylum tricornutum genome and evolutionary origin of diatoms
Source: Sci Rep. 2018 Mar 19;8:4834. doi: 10.1038/s41598-018-23106-x (PMC5859163; doi:10.1038/s41598-018-23106-x)
Supplement: Supplementary file 3 — File S5 [file 41598_2018_23106_MOESM3_ESM.docx]

Integrative analysis of large scale transcriptome data draws a comprehensive landscape of *Phaeodactylum tricornutum* genome and evolutionary origin of diatoms

Achal Rastogi^1^, Uma Maheswari^2^, Richard G. Dorrell^1^, Fabio Rocha Jimenez Vieira^1^, Florian Maumus^3^, Adam Kustka^4^, James McCarthy^5^, Andy E. Allen^5, 6^, Paul Kersey^2^, Chris Bowler^1*^ and Leila Tirichine^1*^

<?xml version="1.0"?>

<!-- Name: Phatr3param-EOULSAN.xml Author: Achal RASTOGI, ENS This file was used as a parameter file to run EOULSAN package for expression analysis.-->

[<analysis>](file:///C:\Users\tirichin\Desktop\dell\MANUSCRIPTS\Phatr3\Scientific%20report\ScientificReports16112017_LT_To%20submit\File_S5.xml)<formatversion>1.0</formatversion><name>my analysis</name><description>All_data_run</description><author>Achal RASTOGI</author>[<constants><parameter>](file:///C:\Users\tirichin\Desktop\dell\MANUSCRIPTS\Phatr3\Scientific%20report\ScientificReports16112017_LT_To%20submit\File_S5.xml)<name>my.constant</name><value>myconstantvalue</value></parameter></constants>[<steps>](file:///C:\Users\tirichin\Desktop\dell\MANUSCRIPTS\Phatr3\Scientific%20report\ScientificReports16112017_LT_To%20submit\File_S5.xml)

<!-- Filter reads -->

[<step skip="**false**">](file:///C:\Users\tirichin\Desktop\dell\MANUSCRIPTS\Phatr3\Scientific%20report\ScientificReports16112017_LT_To%20submit\File_S5.xml)<name>filterreads</name>[<parameters><parameter>](file:///C:\Users\tirichin\Desktop\dell\MANUSCRIPTS\Phatr3\Scientific%20report\ScientificReports16112017_LT_To%20submit\File_S5.xml)<name>illuminaid</name><value/></parameter>[<parameter>](file:///C:\Users\tirichin\Desktop\dell\MANUSCRIPTS\Phatr3\Scientific%20report\ScientificReports16112017_LT_To%20submit\File_S5.xml)<name>trim.length.threshold</name><value>40</value></parameter>[<parameter>](file:///C:\Users\tirichin\Desktop\dell\MANUSCRIPTS\Phatr3\Scientific%20report\ScientificReports16112017_LT_To%20submit\File_S5.xml)<name>quality.threshold</name><value>30</value></parameter>

<!--parameter> <name>readnamestartwith.forbidden.prefixes</name> <value></value> </parameter-->

</parameters></step>

<!-- Map reads -->

[<step skip="**false**">](file:///C:\Users\tirichin\Desktop\dell\MANUSCRIPTS\Phatr3\Scientific%20report\ScientificReports16112017_LT_To%20submit\File_S5.xml)<name>mapreads</name>[<parameters><parameter>](file:///C:\Users\tirichin\Desktop\dell\MANUSCRIPTS\Phatr3\Scientific%20report\ScientificReports16112017_LT_To%20submit\File_S5.xml)<name>mapper</name><value>bowtie</value></parameter>[<parameter>](file:///C:\Users\tirichin\Desktop\dell\MANUSCRIPTS\Phatr3\Scientific%20report\ScientificReports16112017_LT_To%20submit\File_S5.xml)<name>mapperarguments</name><value>-n 2 -l 34 -e 70 -k 2 --best</value></parameter></parameters></step>

<!-- SAM filter -->

[<step skip="**false**">](file:///C:\Users\tirichin\Desktop\dell\MANUSCRIPTS\Phatr3\Scientific%20report\ScientificReports16112017_LT_To%20submit\File_S5.xml)<name>filtersam</name>[<parameters><parameter>](file:///C:\Users\tirichin\Desktop\dell\MANUSCRIPTS\Phatr3\Scientific%20report\ScientificReports16112017_LT_To%20submit\File_S5.xml)<name>removeunmapped</name><value>true</value></parameter>[<parameter>](file:///C:\Users\tirichin\Desktop\dell\MANUSCRIPTS\Phatr3\Scientific%20report\ScientificReports16112017_LT_To%20submit\File_S5.xml)<name>removemultimatches</name><value>true</value></parameter></parameters></step>[<step skip="**false**">](file:///C:\Users\tirichin\Desktop\dell\MANUSCRIPTS\Phatr3\Scientific%20report\ScientificReports16112017_LT_To%20submit\File_S5.xml)<name>expression</name>[<parameters><parameter>](file:///C:\Users\tirichin\Desktop\dell\MANUSCRIPTS\Phatr3\Scientific%20report\ScientificReports16112017_LT_To%20submit\File_S5.xml)<name>counter</name><value>htseq-count</value></parameter>[<parameter>](file:///C:\Users\tirichin\Desktop\dell\MANUSCRIPTS\Phatr3\Scientific%20report\ScientificReports16112017_LT_To%20submit\File_S5.xml)<name>genomictype</name><value>CDS</value></parameter>[<parameter>](file:///C:\Users\tirichin\Desktop\dell\MANUSCRIPTS\Phatr3\Scientific%20report\ScientificReports16112017_LT_To%20submit\File_S5.xml)<name>attributeid</name><value>proteinID</value></parameter>[<parameter>](file:///C:\Users\tirichin\Desktop\dell\MANUSCRIPTS\Phatr3\Scientific%20report\ScientificReports16112017_LT_To%20submit\File_S5.xml)<name>stranded</name><value>no</value></parameter>[<parameter>](file:///C:\Users\tirichin\Desktop\dell\MANUSCRIPTS\Phatr3\Scientific%20report\ScientificReports16112017_LT_To%20submit\File_S5.xml)<name>overlapmode</name><value>union</value></parameter>[<parameter>](file:///C:\Users\tirichin\Desktop\dell\MANUSCRIPTS\Phatr3\Scientific%20report\ScientificReports16112017_LT_To%20submit\File_S5.xml)<name>removeambiguouscases</name><value>false</value></parameter></parameters></step>[<step skip="**false**">](file:///C:\Users\tirichin\Desktop\dell\MANUSCRIPTS\Phatr3\Scientific%20report\ScientificReports16112017_LT_To%20submit\File_S5.xml)<name>normalization</name><parameters/></step>[<step skip="**true**">](file:///C:\Users\tirichin\Desktop\dell\MANUSCRIPTS\Phatr3\Scientific%20report\ScientificReports16112017_LT_To%20submit\File_S5.xml)<name>diffana</name>[<parameters><parameter>](file:///C:\Users\tirichin\Desktop\dell\MANUSCRIPTS\Phatr3\Scientific%20report\ScientificReports16112017_LT_To%20submit\File_S5.xml)<name>disp.est.method</name><value>pooled</value></parameter>[<parameter>](file:///C:\Users\tirichin\Desktop\dell\MANUSCRIPTS\Phatr3\Scientific%20report\ScientificReports16112017_LT_To%20submit\File_S5.xml)<name>disp.est.sharing.mode</name><value>maximum</value></parameter>[<parameter>](file:///C:\Users\tirichin\Desktop\dell\MANUSCRIPTS\Phatr3\Scientific%20report\ScientificReports16112017_LT_To%20submit\File_S5.xml)<name>disp.est.fit.type</name><value>parametric</value></parameter></parameters></step></steps>[<globals>](file:///C:\Users\tirichin\Desktop\dell\MANUSCRIPTS\Phatr3\Scientific%20report\ScientificReports16112017_LT_To%20submit\File_S5.xml)

<!-- Temporary directory to use in local mode -->

[<parameter>](file:///C:\Users\tirichin\Desktop\dell\MANUSCRIPTS\Phatr3\Scientific%20report\ScientificReports16112017_LT_To%20submit\File_S5.xml)<name>main.tmp.dir</name><value>`pwd`/tmp</value></parameter>

<!--parameter> <name>main.local.threads</name> <value>6</value> </parameter-->

</globals></analysis>
